# Insights Into an Unexplored Component of the Mosquito Repeatome: Distribution and Variability of Viral Sequences Integrated Into the Genome of the Arboviral Vector *Aedes albopictus*

**DOI:** 10.3389/fgene.2019.00093

**Published:** 2019-02-12

**Authors:** Elisa Pischedda, Francesca Scolari, Federica Valerio, Rebeca Carballar-Lejarazú, Paolo Luigi Catapano, Robert M. Waterhouse, Mariangela Bonizzoni

**Affiliations:** ^1^Department of Biology and Biotechnology, University of Pavia, Pavia, Italy; ^2^Department of Microbiology & Molecular Genetics, University of California, Irvine, Irvine, CA, United States; ^3^Department of Ecology and Evolution, University of Lausanne and Swiss Institute of Bioinformatics, Lausanne, Switzerland

**Keywords:** mosquitoes, viral integrations, immunity, piRNA pathway, domestication, repeatome

## Abstract

The Asian tiger mosquito *Aedes albopictus* is an invasive mosquito and a competent vector for public-health relevant arboviruses such as Chikungunya (*Alphavirus*), Dengue and Zika (*Flavivirus*) viruses. Unexpectedly, the sequencing of the genome of this mosquito revealed an unusually high number of integrated sequences with similarities to non-retroviral RNA viruses of the *Flavivirus* and *Rhabdovirus* genera. These Non-retroviral Integrated RNA Virus Sequences (NIRVS) are enriched in piRNA clusters and coding sequences and have been proposed to constitute novel mosquito immune factors. However, given the abundance of NIRVS and their variable viral origin, their relative biological roles remain unexplored. Here we used an analytical approach that intersects computational, evolutionary and molecular methods to study the genomic landscape of mosquito NIRVS. We demonstrate that NIRVS are differentially distributed across mosquito genomes, with a core set of seemingly the oldest integrations with similarity to *Rhabdoviruses*. Additionally, we compare the polymorphisms of NIRVS with respect to that of fast and slow-evolving genes within the *Ae. albopictus* genome. Overall, NIRVS appear to be less polymorphic than slow-evolving genes, with differences depending on whether they occur in intergenic regions or in piRNA clusters. Finally, two NIRVS that map within the coding sequences of genes annotated as *Rhabdovirus* RNA-dependent RNA polymerase and the nucleocapsid-encoding gene, respectively, are highly polymorphic and are expressed, suggesting exaptation possibly to enhance the mosquito’s antiviral responses. These results greatly advance our understanding of the complexity of the mosquito repeatome and the biology of viral integrations in mosquito genomes.

## Introduction

The amount and the type of repeated DNA sequences, collectively called the “repeatome,” affect the size, organization and evolution of eukaryotic genomes (Maumus and Quesneville, 2014). Transposable elements (TEs) are the major and most-studied components of the repeatome because of their potential mutagenic effects (Gilbert and Feschotte, 2018). TEs evolve through a “burst and decay model” whereby newly acquired TEs can multiply rapidly in a genome. The “burst” phase is followed by low amplification periods, the “decay” moments, when TEs tend to accumulate mutations and become inactive (Maumus and Quesneville, 2016). In eukaryotes, TE mobilization during germline formation is counterbalanced by the activity of the PIWI-interacting RNA (piRNA) pathway, the most recently identified of three small RNA-based silencing mechanisms (Brennecke et al., 2007; Guzzardo et al., 2013; Gainetdinov et al., 2017). Briefly, Argonaute proteins of the PIWI-subfamily associate with small RNAs of 25-30 nucleotides, called PIWI-interacting RNAs (piRNAs), and together they silence TEs based on sequence complementarity (Tóth et al., 2016). piRNAs arise from genomic regions called piRNA clusters, which contain fragmented sequences of previously acquired TEs.

Unexpectedly, besides TE fragments, piRNA clusters contain sequences from non-retroviral RNA viruses, which produce piRNAs, in the genome of arboviral vectors like the mosquitoes *Aedes aegypti* and *Aedes albopictus* (Arensburger et al., 2011; Olson and Bonizzoni, 2017; Palatini et al., 2017; Whitfield et al., 2017). This observation is in line with recent experimental evidence that extend the role of the piRNA pathway to immunity against viruses in *Aedes* mosquitoes, differently than in *Drosophila melanogaster* (Miesen et al., 2016; Petit et al., 2016) and show that piRNAs from integrated viral sequences are differentially expressed following viral challenge of *Ae. albopictus* (Wang et al., 2018). As such, NIRVS have been proposed as novel immunity factors of arboviral vectors (Olson and Bonizzoni, 2017; Palatini et al., 2017; Whitfield et al., 2017). However, the organization, stability and mode of action of NIRVS in mosquito genomes are poorly understood.

The landscape of viral integrations in the genome of *Ae. aegypti* and *Ae. albopictus* mosquitoes is rather complex. *Aedes* species are rare examples within the animal kingdom because they harbor dozens of NIRVS from different viruses, such as *Flaviviruses* and Mononegavirales, primarily *Rhabdoviruses* and poorly characterized *Chuviruses* (Katzourakis and Gifford, 2010; Fort et al., 2012; Palatini et al., 2017; Whitfield et al., 2017). In all other animals in which NIRVS have been identified, including mammals, birds and ticks, NIRVS appear to be mainly from one viral type and tend to be found in low numbers (<20) (Belyi et al., 2010; Katzourakis and Gifford, 2010; Holmes, 2011; Kryukov et al., 2018). NIRVS identified in the *Ae. aegypti* and *Ae. albopictus* genomes are not homologous, indicating independent integration events. However, NIRVS of both species encompass fragmented viral open reading frames (ORFs). In *Ae. albopictus*, we characterized 32 NIRVS with similarities to *Flaviviruses* (F-NIRVS) and 40 NIRVS similar to *Rhabdoviruses* (R-NIRVS). These NIRVS are enriched in piRNA clusters and within coding sequences (Palatini et al., 2017). Taken together these findings support the hypothesis that NIRVS contribute to host biology. However, because NIRVS have been identified by *in silico* analyses of the currently available *Ae. albopictus* genome assembly, which was built from the DNA of a single pupa of the Foshan strain (Chen et al., 2015) and we verified the overall conservation of NIRVS within this strain (Palatini et al., 2017), their widespread occurrence in wild mosquitoes, whether all NIRVS or some are functionally active elements, and what is the relative importance of each of them, are all still unexplored questions.

Here we addressed the following questions: is the pattern of NIRVS within mosquitoes of the Foshan strain the same as across geographic samples? If the landscape of NIRVS is variable, could NIRVS be co-opted as novel molecular markers for population genetic studies? Does NIRVS age differ depending on their viral origin? How does the LoP of NIRVS compare with that of fast- and slow-evolving mosquito genes?

Using an analytical approach that intersects computational, evolutionary, and molecular approaches we show that NIRVS are a dynamic component of the *Ae. albopictus* repeatome. The landscape of NIRVS is variable within mosquitoes of the Foshan strains and among geographic samples. The LoP of NIRVS is heterogeneous. R-NIRVS appear more widespread and older integrations than those with similarities F-NIRVS. NIRVS annotated in intergenic regions appear more variable than those mapping within piRNA clusters or gene exons. Among NIRVS identified within gene exons, six are fixed and stably expressed, albeit showing different levels of polymorphism and domestication cannot be excluded for AlbRha52 and AlbRha12, which are part of genes annotated as RNA-dependent RNA polymerase and nucleocapsid-encoding genes of *Rhabdovirus*, respectively.

Overall these results greatly advance our understanding of the widespread occurrence of NIRVS in nature. Additionally, a detailed analysis of NIRVS distribution and polymorphism within the *Ae. albopictus* genome paves the way for choosing candidate NIRVS for functional studies.

## Materials and Methods

### Mosquitoes

Mosquitoes of the Foshan strain have been reared at the insectary of the University of Pavia since 2013 (Palatini et al., 2017). Upon arrival in Pavia, mosquitoes were checked for infection using *Flavivirus* degenerate primers (Crochu et al., 2004). No infection was detected. Mosquitoes are reared at 28°C and 70–80% relative humidity with 12/12 h light/dark cycles. Larvae are reared in pans and fed on finely ground fish food (Tetramin, Tetra Werke, Melle, Germany). Adults are kept in 30-cm^3^ cages and allowed access to a cotton wick soaked in 0.2 g/ml sucrose as a carbohydrate source. Adult females are blood-fed using a membrane feeding apparatus and commercially available mutton blood. Sixteen Foshan mosquitoes, eight males and eight females, were sampled and forced in single mating. Progeny from each single mating was collected. DNA was extracted from single individuals, including parents and their progeny, using the DNeasy Blood and Tissue Kit (Qiagen, Hilden, Germany).

### Southern Blotting

Genomic DNA (∼10 mg) from pools of 10–20 adult mosquitoes of the Foshan strain were digested with restriction enzymes (Thermo Scientific, Vilnius, Lithuania) chosen to specifically target individual F-NIRVS and separated on a 0.8% agarose gel. DNA was transferred to nylon membranes (Hybond-N+) (Amersham, Buckinghamshire, United Kingdom) and immobilized by UV irradiation. Random-primed DNA probes (Supplementary Data 1) were labeled with [α-^32^P] dATP/ml and [α-^32^P] dCTP/ml (3,000 Ci/mmol; 1 Ci- 37 GBq) by using the Megaprime labeling kit (Amersham, Buckinghamshire, United Kingdom). Hybridizations were carried out at 65°C.

### Real Time PCR (qPCR) to Test for NIRVS Copy Number

PCR primers were designed using PRIMER3 (Rozen and Skaletksy, 2000) within F-NIRVS to test for their copy number based on real-time PCR (Bubner and Baldwin, 2004; Yuan et al., 2007) (Supplementary Data 2). Reaction mixtures were prepared containing 10 μL QuantiNova Sybr Green PCR Master Mix (Qiagen, Hilden, Germany), 1 μL of each 10 μM primer, and template DNA diluted in distilled H_2_O up to 20 μL total reaction volume. Template genomic DNA used in the reactions was extracted from individual adult mosquitoes following a standard protocol (Baruffi et al., 1995). Real-time PCR reactions were performed in a two-step amplification protocol consisting of 2 min at 95°C, followed by 40 cycles of 95°C for 5 s and 60°C for 10 s. Reactions were run on a RealPlex Real-Time PCR Detection System (Eppendorf, Hamburg, Germany). The single-copy gene *piwi6* (AALF016369) was used as reference after having verified the region of the primers does not harbor variability. F-NIRVS copy numbers were estimated comparing the relative quantification of NIRVS loci with respect to that of the reference genes using the ΔCt method (Pfaffl, 2006), after having verified that the efficiencies of PCR reactions with primers for F-NIRVS and the reference gene were the same. Support for using relative quantification without an internal calibrator came from a preliminary test where we cloned one NIRVS (AlbFlavi4) and we verified that estimates of its copy number by absolute vs. relative quantification were the same.

### qPCR to Estimate NIRVS Expression Levels

Total RNA was extracted using TRIzol (Life Technologies, Madrid, Spain) from pools including 10–20 mosquitoes at different developmental stages such as larvae, pupae, adult males, sugar-fed females and females sampled 48 h after blood feeding. After DNaseI (Sigma-Aldrich, Schnelldorf, Germany) treatment, a total of 100 ng of RNA from each pool was used for reverse transcription using the qScript cDNA SuperMix (Quanta Biosciences, Leuven, Belgium). Expression of the eight N-Gs and always detected in Foshan (i.e., AALF005432, AALF025780, AALF000476, AALF000477, AALF020122, AALF004130, and AALF025779) was quantified using real-time qPCRs following the protocol described above. Expression values were normalized to mRNA abundance levels of the *Ae. albopictus nap* gene (Reynolds et al., 2012) (Supplementary Data 3). The qbase+software (Hellemans et al., 2007) was used to compare expression profiles across samples, and Morpheus^1^ was used to visualize the data.

### Selection of Genes With Slow and High Evolutionary Rates

Orthologous genes across 27 insect species within the Nematocera sub-order were identified in OrthoDB v9.1 (Zdobnov et al., 2016). Levels of sequence divergence were computed for each orthologous group as the average of interspecies amino acid identified normalized to the average identity of all interspecies best-reciprocal-hits, computed from pairwise Smith-Waterman alignments of protein sequences (Supplementary Table 1). We selected the 0.1% of the genes (*n* = 14, number comparable to that of our NIRVS groups) at each tail of the distribution as representative of the conserved and variable categories, the left and right tails respectively. Orthologs of these genes were searched in the *Ae. albopictus* genome (AaloF1 assembly).

### NIRVS in Natural Populations

PCR primers were designed using PRIMER3 (Rozen and Skaletksy, 2000) to test for NIRVS polymorphism in *Ae. albopictus* geographic samples (Supplementary Data 4). Considering the level of NIRVS sequence similarity, their copy number and heterogeneous presence in Foshan mosquitoes, we selected seven F-NIRVS (AlbFlavi2, AlbFlavi4, AlbFlavi8-41, AlbFlavi10, AlbFlavi36, AlbFlavi1, and AlbFlavi12-17) and six R-NIRVS (AlbRha1, AlbRha7, AlbRha14, AlbRha36, AlbRha52, AlbRha85) that gave unambiguous PCR results, have similarities to different viral ORFs and are distributed in different genomic regions including piRNA clusters, intergenic or coding regions. Natural mosquito samples derive from a world-wide collection available at the University of Pavia and previously analyzed with microsatellite markers (Manni et al., 2017). PCR reactions were performed in a final volume of 25 μL using DreamTaq^TM^ Green PCR Master Mix 2x (Thermo Scientific, Vilnius, Lithuania) and the following cycle conditions: 94°C for 3 min, 40 cycles at 94°C for 30 s, 58–60°C for 45 s, 72°C for 1 min, and a final extension at 72°C for 10 min. Amplification products were electrophoresed on 1–1.5% agarose gels and purified using ExoSAP-IT^M^ PCR product Cleanup Reagent (Thermo Scientific, Vilnius, Lithuania). When the NIRVS were detected, at least five amplification products per population per locus were sent to be sequenced by Macrogen (Barcellona, Spain), following the company’s requirements.

Non-retroviral Integrated RNA Virus Sequences alleles were first scored based on their occurrence in each population and their size. A Neighbor-joining tree was built after 1000 bootstrap resampling of the original data set and the calculation of a matrix of shared allele distances (DAS) using POPULATIONS version 1.2.31 (Langella, 1999).

### Estimates of Integration Time

Non-retroviral Integrated RNA Virus Sequences sequences from geographic samples were aligned in Ugene version 1.26.1 (Okonechnikov et al., 2012) with MAFFT (Yamada et al., 2016). Default parameters with five iterative refinements were applied for the alignment. Alignments were manually curated to verify frameshifts, truncations, deletions, and insertions. All positions including gaps were filtered out from the analysis. The following formula was used to estimate the time of integration in years assuming that all mutations are neutral:

$$\mathrm{Mean Mutations/Seq}=\frac{\mathrm{Tot. Obs. Mutations}}{\mathrm{N. Seqs* Seq. Length}}$$

$$\mathrm{Age in Years}=\frac{\mathrm{Mean Mutations/Seq}}{\left(\mathrm{MR* Seq. Length* GpY}\right)}$$

Mutation rate (MR) were assumed to be comparable to those of *D. melanogaster* genes in range 3.5-8.4 × 10^−09^ (Haag-Liautard et al., 2007; Keightley et al., 2009). A range of 4–17 number of generations per year (GpY) was tested considering mosquitoes of temperate or tropical environments (Manni et al., 2017).

### Phylogenetic Inference and Timetrees

Deduced NIRVS protein sequences were aligned with subsets of corresponding proteins from *Flavivirus* and *Rhabdovirus* genomes using MUSCLE (Edgar et al., 2004). The timetrees were generated using the RelTime method (Tamura et al., 2012) after having generated the maximum likelihood tree, with 100 bootstrap replicates. Divergence times for all branching points in the topology were calculated using the maximum likelihood method and implementing the best fitted amino acids substitution model. Phylogenies were estimated in MEGA7 (Kumar et al., 2016). The JTT matrix-based model was used for the L protein of *Rhabdovirus* (Jones et al., 1992). In this case, the estimated log likelihood value was −116005.08. A discrete Gamma distribution was used to model evolutionary rate differences among sites [2 categories (+G, parameter = 0.8331)]. The rate variation model allowed for some sites to be evolutionarily invariable ([+I], 0.21% sites). The analysis involved 49 amino acid sequences. There was a total of 2319 positions in the final dataset. For the G protein of *Rhabdoviruses*, the Whelan and Goldman model was implemented (Whelan and Goldman, 2001). In this case, the estimated log likelihood value was −3719.06. A discrete Gamma distribution was used to model evolutionary rate differences among sites [2 categories (+G, parameter = 2.1095)]. The analysis involved 40 amino acid sequences. All positions with less than 95% site coverage were eliminated. That is, fewer than 5% alignment gaps, missing data, and ambiguous bases were allowed at any position. There was a total of 56 positions in the final dataset. The LG model was used for the NS3 protein of *Flaviviruses* (Le and Gascuel, 2008). In this case, the estimated log likelihood value is −6360.35. A discrete Gamma distribution was used to model evolutionary rate differences among sites [2 categories (+G, parameter = 0.8640)]. The analysis involved 30 amino acid sequences. All positions containing gaps and missing data were eliminated. There was a total of 180 positions in the final dataset. For NIRVS with similarities to the NS5 protein of *Flaviviruses* the JTT matrix-based model was used (Jones et al., 1992). The estimated log likelihood value of the topology shown was −26019.44. A discrete Gamma distribution was used to model evolutionary rate differences among sites [2 categories (+G, parameter = 1.0058)]. The rate variation model allowed for some sites to be evolutionarily invariable ([+I], 10.15% sites). The analysis involved 33 amino acid sequences. There was a total of 984 positions in the final dataset. In each case, trees were drawn to scale, with branch lengths measured in the relative number of substitutions per site. The coding sequences for proteins of the Potato Yellow Dwarf virus (PYDV) were used as outgroup for trees of R-NIRVS, considering PYDV belongs to the highly divergent *Nucleorhabdovirus* genus (Dietzgen et al., 2017). To derive the genealogy of F-NIRVS, outgroups were protein sequences from Tamana Bat Virus (TABV) (de Lamballerie et al., 2002).

### Bioinformatic Pipeline to Study the Polymorphisms of NIRVS, Fast- and Slow-Evolving Genes

Whole genome sequencing data of 16 singly sequenced (i.e., single-sequenced mosquitoes or SSMs) as previously described (Palatini et al., 2017) was used for the analyses of NIRVS polymorphism. NIRVS presence in a sample was established imposing a more stringent criteria than previously used in Palatini et al. (2017). Here to the “minimum of five reads of depth of coverage,” we added a minimum of 30 consecutive nucleotides with that depth of coverage (Supplementary Table 2). This more stringent criteria resulted in a difference of one in the number of NIRVS called as absent (AlbRha43). We molecularly validated bioinformatic predictions based on this criterion (Supplementary Figure 1). The ratio between the number of R-NIRVS present in a sample and the total R-NIRVS of Foshan (40) was used to estimate R-NIRVS prevalence. The same calculation was done for F-NIRVS. The polymorphism of NIRVS and that of selected FGs and slow-evolving genes (i.e., SGs) was then estimated using a custom pipeline organized into different steps. In the first step, the DepthOfCoverage function of the GATK tool (Mckenna et al., 2010) is used to evaluate the coverage of the region of interest limiting to reads with Phred mapping quality greater than 20. Following read coverage analyses, four different Variant Callers i.e., GATK UnifiedGenotyper (Mckenna et al., 2010), Freebayes (Garrison and Marth, 2012), Platypus (Rimmer et al., 2014), and Vardict (Lai et al., 2016), were implemented to identify SNPs and INDELs within the regions of interest. The search of SNPs and INDELS by different variant callers allowed to increase the pool of variants and reduce the number of false positive. Custom scripts were then used to filter data, retain only variants having allele frequency higher than 0.1 or variants called by at least two programs. The LoP of the region of interest was calculated as the total number of SNPs and INDELs identified averaged based on its length.

Follow-up statistical analyses were computed and visualized in R studio (RStudio Team, 2015). RStudio: Integrated Development for R. RStudio, Inc. (Boston, 2015). The Kolmogorov–Smirnov test was used to test the significance of the difference of LoP distributions of NIRVS, RNAi genes (R-Gs), N-Gs and FGs with respect to that of SGs (Supplementary Table 3). SG LoP was the median of the LoPs of the tested SGs. The threshold of significance was adjusted with the Bonferroni correction and loci were separated according to the adjusted significance of the test. Results of ratio between the LoP of each locus and the median LoP of SGs (fold change [FC]) that were different from 0 were visualized in a volcano plot. For each locus, FC was calculated as the ratio of the median LoP of the locus and that of the SG. The hypergeometric test was applied to test whether the group of NIRVS always identified across SSMs was enriched in (1) F- or R-NIRVS; (2) any viral ORFs; (3) NIRVS shorter or longer than 500 bp; (4) NIRVS mapping in exons, piRNA clusters or intergenic regions.

### Search for Novel Viral Integrations

Sequences supported by the presence of soft-clipped reads were molecularly tested by PCR assays using DNA from individual mosquitoes of the Foshan strain (Supplementary Data 5). The Vy-PER pipeline (Forster et al., 2015) was applied to WGS data from the 16 SSMs to search for viral integrations that had not been previously identified in genome of the Foshan strain (AaloF1 assembly). Vy-PER was run using 540 viral genomes from VIPERdb (Carrillo-Tripp et al., 2009), including species of the Togaviridae, Flaviviridae, Bunyaviridae, Rhabdoviridae, Orthomyxoviridae, Reoviridae, Bornaviridae, Filoviridae, Nyamiviridae, Paramyxoviridae families. Paired-end reads identified by Vy-PER in which one read maps to the reference mosquito genome (i.e., AaloF1) and its pair maps to one of the tested viral genomes were manually inspected. Candidates including low complexity sequence (i.e., sequence showing more than 80% in mono- and di-nucleotides) or with viral portion shorter than 50 nucleotides were considered false positive and were filtered out.

## Results

We use read depth of coverage and variant calling tools to study NIRVS (Non-retroviral Integrated RNA Virus Sequences) widespread occurrence and their polymorphism within the genomes of mosquitoes of the Foshan strain and to look for novel viral integrations. Additionally, we studied the distribution of a selected subset of NIRVS in geographic samples.

### NIRVS Are Variably Distributed in SSMs

**FIGURE 1:**
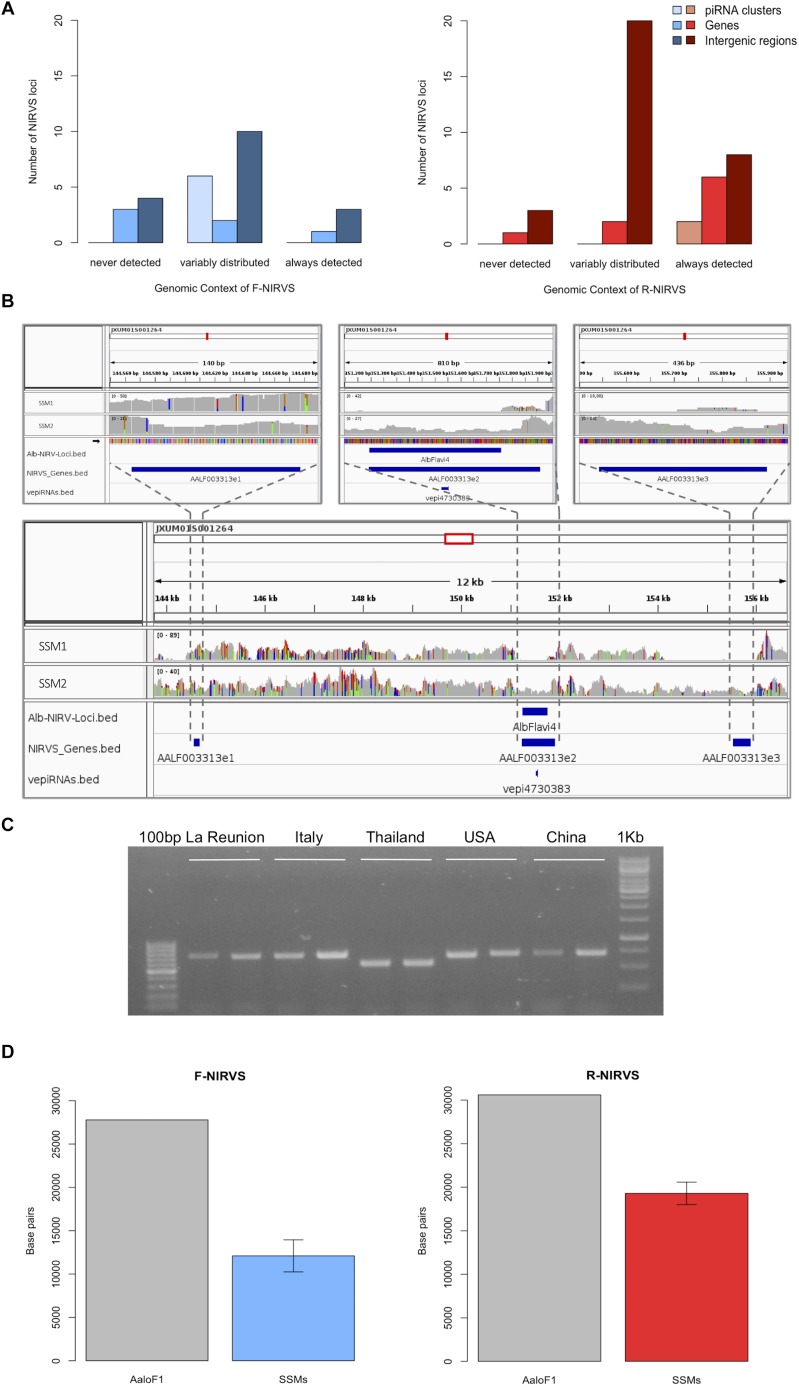
NIRVS are variably distributed in SSMs. **(A)** Number of *Flavivirus* (F-NIRVS) and *Rhabdovirus* (R-NIRVS) loci mapping within genes, piRNA clusters or intergenic regions, classified on the basis of read-coverage across SSMs. **(B)** IGV screen shot showing read-coverage at AALF003313 in SSM1 and SSM2. Positions of the three AALF003313 exons, AlbFlavi4 and vepi4730383 are indicated by blue bars. **(C)** PCR amplification of AALF003313 exon2 in ten *Ae. albopictus* geographic samples. **(D)** F-NIRVS and R-NIRVS loci occupancy in the 16 single-sequenced mosquitoes (SSMs) of the Foshan strain is about half of that expected based on the annotated sequences of the reference genome assembly (AaloF1). F-NIRVS are in blue, R-NIRVS are in red.

We used the sequenced genomes of 16 mosquitoes (i.e., single-sequenced mosquitoes or SSMs) (Palatini et al., 2017) and we compared their NIRVS pattern with the list of viral integrations characterized from the Foshan genome assembly (AaloF1). Eleven NIRVS (i.e., AlbFlavi19, AlbFlavi31, AlbFlavi32, AlbFlavi33, AlbFlavi38, AlbFlavi39, AlbFlavi40, AlbRha43, AlbRha79, AlbRha80, AlbRha95) were absent in all 16 SSMs (Supplementary Table 2). A total of 20 NIRVS were found in all SSMs, with a statistical enrichment for NIRVS with similarities to *Rhabdovirus* (R-NIRVS) (Hypergeometric test, *p* = 0.022) and NIRVS mapping in gene exons (Hypergeometric test, *p* = 0.006) (Figure 1A). This “core” of 20 NIRVS included R-NIRVS identified within the coding sequence of genes (i.e., AlbRha12, AlbRha15, AlbRha28, AlbRha52, AlbRha85 and AlbRha9) and piRNA clusters (i.e., AlbRha14 and AlbRha36). Conversely, NIRVS with similarities to *Flaviviruses* (F-NIRVS) were variably distributed among SSMs. Of note is AlbFlavi4, a 512bp sequence with similarity to the capsid gene of *Aedes flavivirus* (Palatini et al., 2017). AlbFlavi4 is annotated within the second exon of AALF003313 and is also included in piRNA cluster 95 (Liu et al., 2016). AlbFlavi4 produces vepi4730383, a piRNA that is upregulated upon dengue infection (Wang et al., 2018). In SSMs and *Ae. albopictus* geographic samples, variants were identified for AALF003313, only one of which includes AlbFlavi4 (Figure 1B,C).

Overall, mean base pairs (bp) occupied by F-NIRVS and R-NIRVS are 12095 and 19293 bp, respectively (Figure 1D). Taken together, these results demonstrate that, with an average genome occupancy of 31389 bp, NIRVS represent quantitatively a limited fraction of the mosquito repeatome. However, the enrichment of NIRVS in piRNA clusters (Palatini et al., 2017) and the fact that the pattern of NIRVS is variable in host genomes support the hypothesis that NIRVS are a dynamic component of the repeatome.

### NIRVS Distribution in Geographic Populations

**FIGURE 2:**
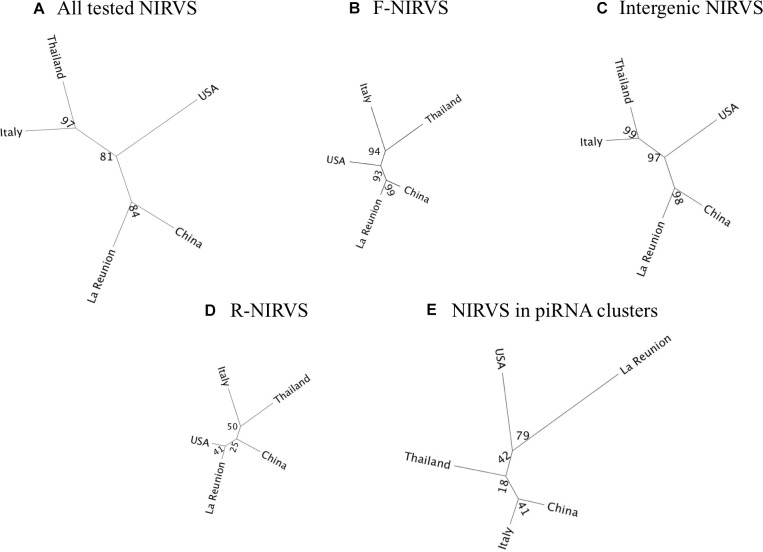
Phylogeographic distribution of NIRVS. Genetic relationships among five mosquito populations shown by a Neighbor-joining trees based on shared-allele distance using data from all 13 genotyped NIRVS **(A)**, only F-NIRVS **(B)**, NIRVS mapping in intergenic regions **(C)**, only R-NIRVS **(D)** and NIRVS mapping in piRNA clusters **(E)**. Bootstrap values are shown at the corresponding nodes.

To verify if NIRVS are variably distributed in natural samples besides in the Foshan strain, we choose seven F-NIRVS (AlbFlavi2, AlbFlavi4, AlbFlavi8-41, AlbFlavi10, AlbFlavi36, AlbFlavi1, and AlbFlavi12-17) and six R-NIRVS (AlbRha1, AlbRha7, AlbRha14, AlbRha36, AlbRha52, AlbRha85) based on their unique occurrence in different regions of the mosquito genome and their similarity to various viral ORFs. AlbRha52 and AlbRha85 are annotated as unique exons of AALF020122 and AALF004130, respectively. We tested the presence of these NIRVS in native (China and Thailand), old (La Reunion Island) and new (United States and Italy) *Ae. albopictus* populations (Manni et al., 2017). NIRVS alleles were differentially distributed across geographic populations so that a tree built from a matrix of shared-allele distances (DAS) proved able to differentiate mosquito populations in accordance with the historical records of *Ae. albopictus* invasive process when considering all thirteen NIRVS, only F-NIRVS or NIRVS mapping in intergenic regions (Figure 2A–C and Supplementary Data 6). On the contrary, when data from exclusively R-NIRVS or NIRVS identified in piRNA clusters, were analyzed, bootstrap values differentiating populations were below 50% (Figure 2D,E). This result agrees with the observation that the higher abundant R-NIRVS are also more prevalent than F-NIRVS.

### R-NIRVS Appear to Be Older Integrations Than F-NIRVS

**FIGURE 3:**
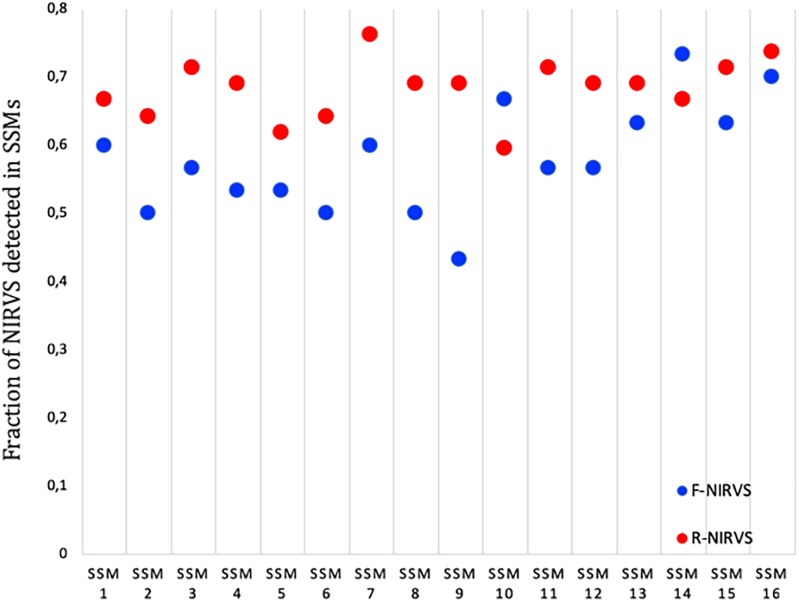
Prevalence of R-NIRVS (red) and F-NIRVS (blue) in each SSMs. Prevalence was calculated in each sample as the ration between detected NIRVS and annotated NIRVS for both R- and F-NIRVS.

**FIGURE 4:**
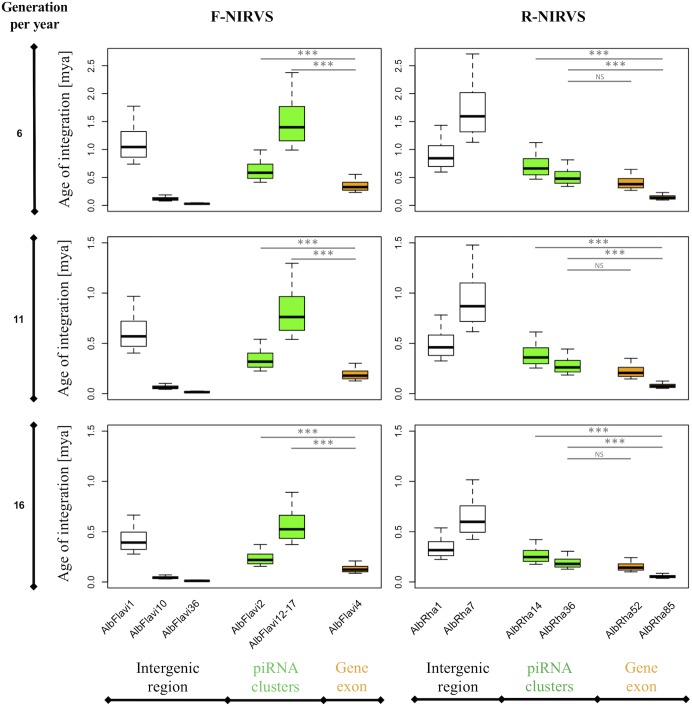
NIRVS integration times. Boxplots showing the integration times for the NIRVS whose variability was studied across five geographic populations. Estimates are based on the *D. melanogaster* mutation rate, i.e., 3.5–8.4 × 10^−9^ per site per generation (Haag-Liautard et al., 2007; Keightley et al., 2009), and a range of generations per year between 6 and 16 to include mosquitoes from temperate and tropical regions (Manni et al., 2017). NIRVS of piRNA clusters are statistically older than NIRVS mapping in gene exons (ANOVA, ^∗∗∗^*P* < 0.001).

The higher prevalence of R-NIRVS with respect to F-NIRVS suggests R-NIRVS are older integrations (Figure 3). To verify this hypothesis, we sequenced alleles of NIRVS identified in the five tested populations and we estimated integration times, assuming comparable mutation rates between *Ae. albopictus* and *D. melanogaster*, that is 3.5–8.4 × 10^−9^ per site per generation (Haag-Liautard et al., 2007; Keightley et al., 2009), and a range of generations per year between 4 and 17, accounting for mosquitoes from temperate and tropical environments, respectively (Manni et al., 2017). Under these conditions, R-NIRVS integrated between 36 thousand and 2.7 million years ago (mya) and F-NIRVS between 7.4 thousand and 2.4 mya (Figure 4). This large window supports the conclusion that integration of viral sequence is a dynamic process occurring occasionally at different times. As shown in Figure 4, estimates of integration times varied greatly depending on the genomic context of NIRVS. NIRVS annotated within gene exons appear statistically more recent than NIRVS of piRNA clusters (ANOVA, ^∗∗∗^*P* < 0.001). Besides reflecting a different integration time, this result is consistent with the hypothesis that integrations within exons are under rapid evolution, a hallmark of domestication (Frank and Feschotte, 2017).

Additionally, we tested the genealogy of R-NIRVS and F-NIRVS in comparison to circulating *Rhabdoviruses* and *Flaviviruses*. Relative timetrees were generated for (i) F-NIRVS and corresponding NS3 and NS5 viral proteins from representative *Flaviviruses*, and (ii) R-NIRVS and corresponding L and G proteins of representative *Rhabdoviruses*. Timetrees showed shorter divergence times between F-NIRVS and viral proteins than R-NIRVS and viral proteins. This clearly indicated multiple integration events and a tendency of R-NIRVS to be older integrations (Supplementary Figure 2).

### NIRVS Are Heterogeneously Polymorphic at the Sequence Level, With the Majority Being Less Variable Than Slow-Evolving Genes

We selected genes having low and high evolutionary rates in *Ae. albopictus* and we compared their levels of polymorphism (LoP) with that of NIRVS in WGS data from our 16 SSMs. LoP was evaluated as the ratio between the number of total mutations (SNPs and INDELs) found in the locus and its length. We expanded the analyses to include also R-Gs, for which intraspecific rapid evolution has been observed in *Ae. aegypti* (Bernhardt et al., 2012), and the 13 N-Gs in their coding sequence or UTRs (Palatini et al., 2017).

**FIGURE 5:**
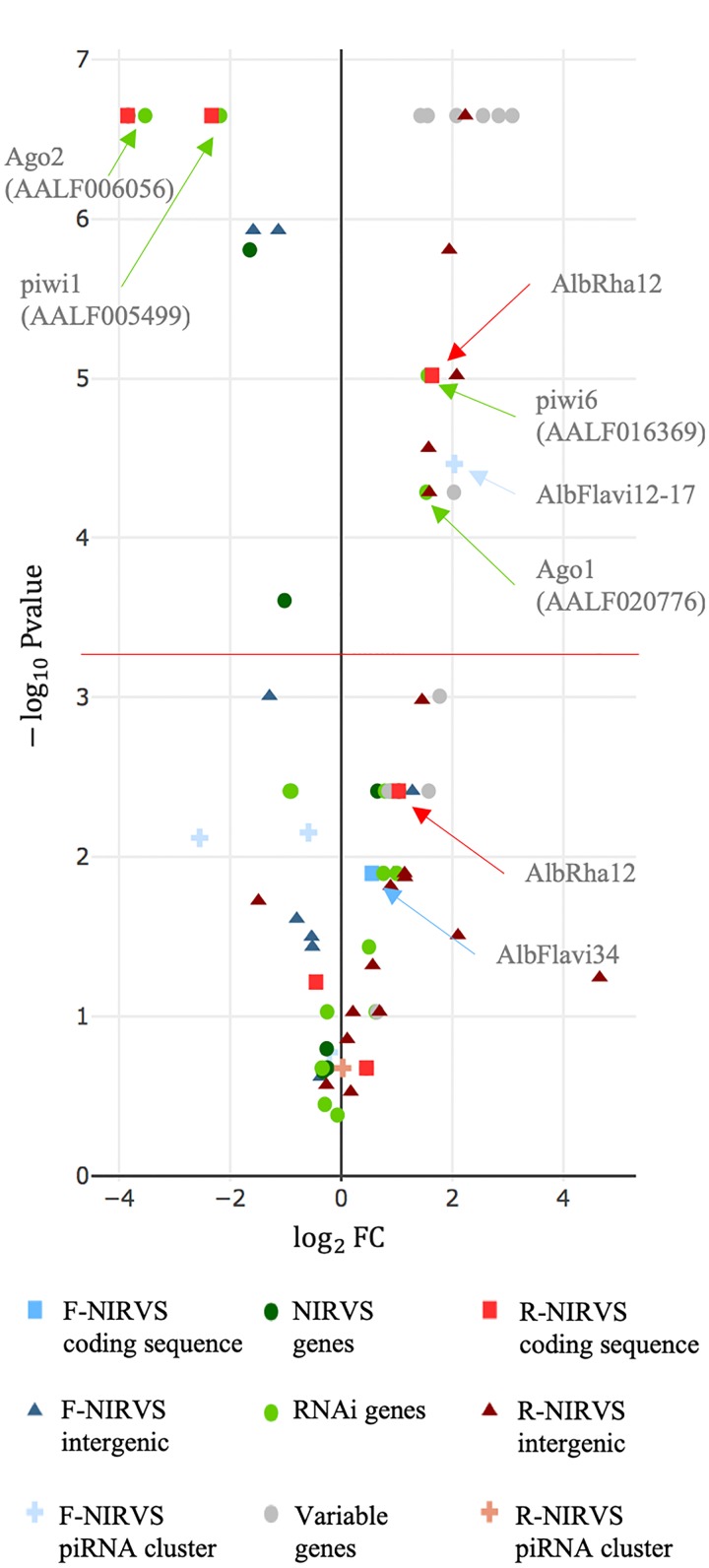
NIRVS polymorphism. Volcano plot showing LoP comparison between SGs and NIRVS, genes encompassing NIRVS (Palatini et al., 2017), genes of the RNAi pathway and FGs. Entities with LoPs statistically different than that of conserved genes are above the red line [adjusted significance of the test (–log10 0.05/99 = 3.32)]. Entities on the left side of the panel (log2FC < 0) have smaller LoP than conserved genes. The opposite for entities on right side of the panel (log2FC > 0).

Estimates of gene evolutionary rates were derived from comparisons of levels of protein sequence divergence within groups of orthologous genes across 27 insect species of the Nematocera sub-order (Supplementary Table 1). The first and last 0.1% of the genes from the evolutionary rate distribution were selected as slow and fast evolving genes, respectively, and their single-copy orthology status with respect to *Ae. albopictus* was verified. We did not select genes based on their length because of the wide length range of NIRVS, which includes partial viral ORFs of between 151 and 3206 bp (Palatini et al., 2017). SGs that met the above criteria include genes with hypothetical protein transporter or vesicle-mediated transport activity (*i.e.*, AALF003606, AALF014156, AALF014287; AALF014448; AALF004102), structural activity (AALF005886, annotated as tubulin alpha chain), signal transducer activity (AALF026109), protein and DNA binding activity (AALF027761, AALF028431), SUMO transferase activity (AALF020750), the homothorax homeobox encoding gene AALF019476, the tropomyosin invertebrate gene (AALF0082224), the Protein yippee-like (AALF018378) and autophagy (AALF018476). FGs include genes with unknown functions (AALF004733, AALF009493, AALF009839, AALF012271, AALF026991, AALF014993, AALF017064, AALF018679), proteolysis functions (AALF010748) a gene associated with transcriptional (AALF022019), DNA-binding (AALF019413, AALF024551), structural (AALF028390) and proteolytic (AALF010877) activities. Median LoP of SGs within mosquitoes of the Foshan strain is 0.0071, a value higher than that observed across 63.3% of the detected NIRVS (Supplementary Figure 3). Eleven out of fourteen FGs were more variable than SGs, with seven appearing also statistically more polymorphic than SGs (Kolmogorov-Smirnov test, ^∗^*P* < 0.05) (Figure 5 and Supplementary Table 3). This result further supports our selection of SGs and FGs.

Genes of the RNAi pathway are heterogeneously polymorphic (Figure 5), with *Ago1* (AALF020776) and *piwi6* (AALF016369) being statistically more polymorphic than SGs; the opposite result was obtained for *piwi1* and *3* (AALF005499, AALF005498), and *Ago2* (AALF006056) (Figure 5). LoP of NIRVS is heterogeneous both among SSMs and. NIRVS identified within piRNA clusters (Liu et al., 2016) are all less polymorphic than SGs, with the exception of AlbFlavi12-17 that has a median LoP value of 0.0258. This large LoP may be due by the fact that AlbFlavi12-17 is composed of four small viral sequences nested one next to the other (Palatini et al., 2017). Unlike NIRVS from piRNA clusters, NIRVS spanning gene exons are more heterogeneous; three (i.e., AlbFlavi34, AlbRha12, and AlbRha52) have LoP values higher than those of SGs, while others (*i.e.*, AlbFlavi24, AlbRha28, AlbRha85) are less polymorphic than SGs. AlbFlavi24, AlbFlavi34, AlbRha12, and AlbRha28 are annotated as the only exons of AALF023281, AALF005432, AALF025780, AALF000478, respectively.

**Table 1:** Characteristics of genes with NIRVS in their coding sequence.

Gene ID	NIRVS	Viral ORF	PfamID	Median LoP
AALF000476^a^	AlbRha15	*Rhabdovirus* nucleocapsid protein	PF00945	0.0086
AALF000477^a^	AlbRha18	*Rhabdovirus* nucleocapsid protein	PF00945	0.0052
AALF000478^a,c^	AlbRha28	*Rhabdovirus* nucleocapsid protein	PF00945	0.0004
AALF025780^a^	AlbRha12	*Rhabdovirus* nucleocapsid protein	PF00945	0.0129
AALF025779^a^	AlbRha9	*Rhabdovirus* nucleocapsid protein	PF00945	0.0031
AALF004130^b^	AlbRha85	*Rhabdovirus* RNA dependent RNA polymerase	PF00946	0.0020
AALF020122^b^	AlbRha52	*Rhabdovirus* RNA dependent RNA polymerase	PF00946	0.0196
AALF005432	AlbFlavi34	*Flavivirus* NS2A, NS2B, NS3	PF00949, PF00271, PF07652	0.0099

*^a^Paralogous genes; ^b^paralogous genes; ^c^no expression data.*

### NIRVS Identified Within Coding Sequences Are Expressed

**FIGURE 6:**
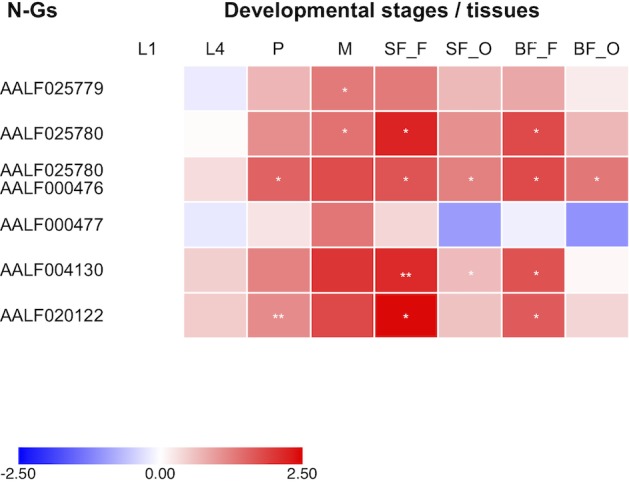
Expression of NIRVS mapping in coding sequences. Heatmap of the expression profiles of genes with NIRVS in the coding sequence (N-Gs) across developmental stages and body tissues (L1-L4: 1st-4th instar larvae; P: pupae; M: male whole body; SF_F/BF_F: sugar/blood fed female whole body; SF_O/BF_O: ovaries from sugar/blood fed females). Color key expresses the log10-fold change relative to larva 1st instar (calibrator). Asterisks indicate significant differences in transcript abundances (Unpaired two-tailed *t*-tests, ^∗^*P* < 0.05, ^∗∗^*P* < 0.01).

The observed LoP for AALF020122 with AlbRha52, AALF025780 with AlbRha12 and AALF005432 with AlbFlavi34 is analogous to that of rapidly evolving genes, suggesting co-option for immunity functions (Frank and Feschotte, 2017). Because domestication of exogenous sequences is a multi-step process, including persistence, immobilization and stable expression of the newly acquired sequences besides rapid evolution (Joly-Lopez and Bureau, 2018), we analyzed the distribution and expression pattern of these genes. Expression analyses were extended to all other N-Gs (AALF025779 with a unique exon containing AlbRha9, AALF000476 with a unique exon corresponding to AlbRha15, AALF000477, and AALF004130 in which the unique exons are contained within AlbRha18 and AlbRha85, respectively) that are fixed within the Foshan strain, but have LoP levels comparable to or lower than those of conserved genes (Figure 5). AlbFlavi34 had been previously studied and showed to be expressed in pupae and adult males more than in larvae (Palatini et al., 2017). Genes with NIRVS (N-Gs) form two groups of paralogs, with similarity to the *Rhabdovirus* RNA-dependent RNA polymerase (RdRPs) and the nucleocapsid-encoding gene, respectively (Table 1). As shown in Figure 6, apart from AALF00477, all other genes are expressed throughout *Ae. albopictus* development with a similar profile, but at different levels. None of the genes showed sex-biased expression or tissue-specific expression in the ovaries; on the contrary highest expression was observed in sugar- and blood-fed females.

### Additional NIRVS Variants Are Found in the Genome of Foshan Mosquitoes

**FIGURE 7:**
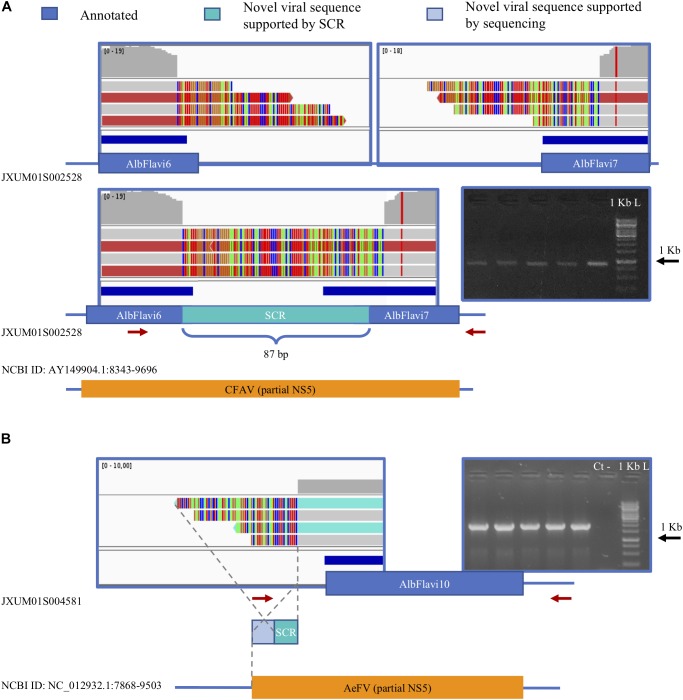
Soft clipped reads (SCR) support novel arrangements and longer than annotated viral integrations. **(A)** AlbFlavi6 and AlbFlavi7 are annotated on the same contig, but 5000 bp apart from each other. Soft-clipped reads (in light blue) and PCR experiments support their contiguity, with a unique ORF with similarity to the *Flavivirus* NS5. **(B)** A sequence of 212 bp extending AlbFlavi10 on its left side was identified investigating SCRs and confirmed by further sequencing. Red arrows indicate primer positions.

We verified the presence of novel NIRVS alleles by investigating soft-clipped reads. Soft-clipped reads support the contiguity of AlbFlavi6 and AlbFlavi7, that were annotated in separated regions of the same contig (Figure 7A). This newly resolved arrangement revealed the existence of a viral ORF of 1191 bp, corresponding to a partial non-structural protein 5 (NS5) of *Flaviviruses*. Additionally, soft-clipped reads supported longer than annotated alleles in AlbFlavi10, AlbFlavi2 and AlbRha4 (Figure 7B). We further looked for the presence of novel viral integrations using Vy-PER (Forster et al., 2015). No viral integrations different than the ones identified *in silico* from the Foshan genome (Palatini et al., 2017) were found in the 16 SSMs.

## Discussion

Repetitive DNA is a major source of genome variability and there are also examples of repetitive sequences being co-opted for cellular functions (Gilbert and Feschotte, 2018). Besides having an impact on the evolution, organization and behavior of eukaryotic genomes, variations in repeat sequences or their copy numbers have been exploited for taxonomic and phylogenetic studies (Djadid et al., 2006; Wang-Sattler et al., 2007; Lammers et al., 2017). Therefore, knowledge of the repeatome assists in understanding the plasticity of eukaryotic genomes and may provide markers for population genetic studies (Goubert et al., 2016, 2017). Repetitive DNA represents 68% of the genome of *Ae. albopictus* and include dozens of still-poorly characterized sequences from non-retroviral RNA viruses (Chen et al., 2015; Palatini et al., 2017). Here we studied the widespread occurrence of NIRVS in relation to the geographic distribution of the species and the variability of NIRVS in comparison to that of mosquito genes showing slow and high evolutionary rates. We clearly show that the landscape of viral integrations is variable within and across geographic populations, with a core set of seemingly the oldest integrations from *Rhabdoviruses*. Additionally, the polymorphism of viral integrations is heterogeneous and depends primarily on their location within the genome. Overall, results of this study emphasize the complexity of the composition and structure of the mosquito repeatome and provide an objective strategy to identify viral integrations that most probably affect mosquito biology.

### Biological Significance of NIRVS Variable Genomic Landscape and Their Polymorphism in SSMs

The landscape of viral integrations is variable among SSMs, longer than annotated alleles are identified through the analyses of soft-clipped reads, but no additional viral sequences, different than the ones characterized from the Foshan-based genome assembly, are found in SSMs. NIRVS are considered rare events following viral infections. In *Aedes* spp. mosquitoes and mosquito cells short segments of cDNA of viral origin (vDNA) are synthetized upon infection with arboviruses of different genera (*i.e., Flavivirus*, *Alphavirus* and *Bunyavirus*) by the reverse transcriptase of endogenous retrotransposons (Goic et al., 2016; Nag and Kramer, 2017). These vDNAs are composed of fragmented viral sequences, from different regions of the viral genome, next to sequences of TEss (Goic et al., 2016; Nag and Kramer, 2017). Because the composition of vDNAs is analogous to that of NIRVS, vDNAs have been proposed to be the substrate for NIRVS (Olson and Bonizzoni, 2017; Palatini et al., 2017). The SSMs analyzed in this study are from the Foshan strain. The Foshan strain derives from mosquitoes collected in the Chinese city of Foshan in the early ‘1980 and have since been kept in laboratory settings with no viral exposure (Chen et al., 2015). Under this scenario, the absence of novel viral integrations in SSMs is not unexpected. However, the identification of a variable landscape among SSMs with a core set of NIRVS, which is enriched for integrations with similarity to *Rhabdoviruses* and NIRVS mapping in coding sequences, is significant because it demonstrates that viral integrations are a dynamic component of the repeatome and not all viral integrations are dispensable genomic elements. Interestingly, when compared to fast- and slow-evolving mosquito genes, NIRVS polymorphism was not homogeneous. NIRVS identified within piRNA clusters were less polymorphic than SGs. Selection constraints on sequences within piRNA clusters have been previously identified in both flies and mice (Chirn et al., 2015). This is despite piRNAs have an incredible sequence diversity and their biogenesis and processing do not appear to be linked to common sequences or structural motifs (Huang et al., 2017). In *D. melanogaster*, piRNA clusters are dynamic loci and their composition has been linked to their regulatory abilities. For instance, the ability of the *D. melanogaster* master piRNA locus *flamenco* to control transposons such as *gypsy*, *ZAM* and *Idefix* was shown to be dependent on frequent chromosomal rearrangements, loss or gain of fragmented TE sequences (Zanni et al., 2013; Guida et al., 2016). Additionally, variations in the composition of subtelomeric piRNA clusters were observed upon adaptation to laboratory conditions of *D. melanogaster* wild collected flies (Asif-Laidin et al., 2017). Importantly, structural differences in subtelomeric piRNA clusters did not impair host genome integrity and occurred with the maintenance of conserved groups of sequences, which could be alternatively distributed among different strains (Asif-Laidin et al., 2017). Data on the geographic distribution of NIRVS mapping in piRNA clusters studied here (i.e., AlbFlavi2, AlbFlavi4, AlbFlavi12-17, AlbRha14, and AlbRha36) show a situation analogous to that identified with TE fragments of the *flamenco* locus in *D. melanogaster*. On this basis, it is tempting to propose that the analogy between *D. melanogaster* and *Ae. albopictus* in the dynamic composition of piRNA clusters extends to their function so that the pattern of viral integrations within piRNA clusters influence mosquito susceptibly to viral infection. If proven, this hypothesis may help explain the observed variability in vector competence across mosquito populations and could be adapted into novel genetic-based strategies of vector control.

Among NIRVS encompassing gene exons, three appeared more variable than FGs and are also expressed; two of these (AlbRha52 and AlbRha12) are also persistent suggesting exaptation (Joly-Lopez and Bureau, 2018). AlbRha52 and AlbRha12 have similarity to the RdRPs and nucleocapsid-encoding genes of *Rhabdovirus*, respectively. RdRPs are ancient enzymes, essential for RNA viruses (de Farias et al., 2017). While the existence of RdRP genes in insects is still debated, cellular RdRP activity has been observed in plants, fungi and *Caenorhabditis elegans* in association with RNA silencing functions (Zong et al., 2009; de Farias et al., 2017; Pinzon et al., 2018). An RdRP of viral origin was recently described in a bat species of the *Eptesicus* clade (Horie et al., 2016) and exaptation of a viral nucleocapsid gene was shown in Afrotherians (Kobayashi et al., 2016). On this basis, further experiments to characterize the functions of the *Ae. albopictus* genes AALF020122 and AALF025780 are on-going.

### Biological Significance of NIRVS Variable Genomic Landscape in Geographic Populations

To start gaining insights into the natural widespread occurrence of NIRVS, a set of 13 viral integrations representative of both R- and F-NIRVS and mapping within piRNA clusters, intergenic regions and gene exons were selected and both their occurrence and their sequence polymorphism was analyzed in mosquitoes from five geographic populations. Populations were selected following the invasion history of *Ae. albopictus* out of its native home range in south East Asia and included samples from China, Thailand, La Reunion island and newly colonized areas such as Italy and United States. Distributions of NIRVS in these populations was consistent with results from SSMs as R-NIRVS were more frequently detected than F-NIRVS. Additionally, R-NIRVS appeared on overage older integrations than F-NIRVS.

The difference in the number and age of the integration events among sequences from *Rhabdoviruses* and *Flaviviruses* is intriguing because Mononegavirales, including *Rhabdoviruses*, are considered evolutionary more recent than *Flaviviridae* (Koonin et al., 2015). The *Rhabdovirus* genus contains viruses that are extremely variable in both their genomic organization and host preferences, with viruses infecting vertebrates, invertebrates and plants (Dietzgen et al., 2017; Geoghegan et al., 2017). Additionally, *Rhabdoviruses* have been shown to frequently transfer horizontally among host species based on their ecological and geographic proximity (Geoghegan et al., 2017). Thus, the ecological diversity and the wide geographic distribution range of *Rhabdoviruses* may favor their integrations into mosquito genomes. Alternatively, the promiscuous nature of *Rhabdoviruses* with frequent horizontal transfers could select for the emergence of generalist protection mechanisms, of which integrations could be part of.

The variable landscape of NIRVS across geographic populations should be interpreted with caution. The rapid global invasion of *Ae. albopictus* from South-East Asia, which happened over the past 50–60 years, was human-mediated and occurred through the movement of propagules (Manni et al., 2017), creating a situation of genetic admixture. Mosquito populations from newly invaded areas, such as Italy and United States, lack isolation by distance and appear genetically mixed (Kotsakiozi et al., 2017; Manni et al., 2017; Maynard et al., 2017). The occurrence of frequent bottlenecks followed by interbreeding can partly explain the variable NIRVS landscape observed here. However, the enrichment for R-NIRVS, the variable distribution of NIRVS within piRNA clusters and their heterogenous polymorphism indicate that evolutionary forces other than genetic drift and gene flow have played a role in the distribution of NIRVS and suggests a multifaceted impact of NIRVS on mosquito physiology.

## Data Availability Statement

Whole Genome Sequencing data alignments have been deposited to the SRA archive under accession number from SAMN09759672 to SAMN09759687.

## Author Contributions

MB and EP conceived and designed the study, analyzed the data, and drafted the manuscript. EP and RW contributed to bioinformatic analyses, analyzed the results, and revised the manuscript. FS, FV, PC, and RC-L collected and analyzed molecular data and revised the manuscript. All authors read and approved the final manuscript.

## Conflict of Interest Statement

The authors declare that the research was conducted in the absence of any commercial or financial relationships that could be construed as a potential conflict of interest.

## Footnotes

1 https://software.broadinstitute.org/morpheus

## Abbreviations

FC: fold-change
FGs: fast-evolving genes
F-NIRVS: NIRVS with similarity to *Flaviviruses;* LoP, level of polymorphism
N-Gs: genes harboring NIRVS
NIRVS: non-retroviral integrated RNA virus sequences
R-Gs: Genes of the RNAi pathway
R-NIRVS: NIRVS with similarity to *Rhabdoviruses;* SGs, slow-evolving genes
SSM: single-sequenced mosquitoes
