# Supplementary material for: Insights Into an Unexplored Component of the Mosquito Repeatome: Distribution and Variability of Viral Sequences Integrated Into the Genome of the Arboviral Vector Aedes albopictus
Source: Front Genet. 2019 Feb 12;10:93. doi: 10.3389/fgene.2019.00093 (PMC6379468; doi:10.3389/fgene.2019.00093)

Supplementary Material

Insights into an unexplored component of the mosquito repeatome: distribution and variability of viral sequences integrated into the genome of the arboviral vector Aedes albopictus

Elisa Pischedda^1^, Francesca Scolari^1^, Federica Valerio^1^, Rebeca Carballar-Lejarazú^2^, Paolo Luigi Catapano^1^, Robert M. Waterhouse^3^ and Mariangela Bonizzoni^1*^

*** Correspondence:** Mariangela Bonizzoni: [m.bonizzoni@unipv.it](mailto:m.bonizzoni@unipv.it)

Supplementary Data

# List of probes used for Southern Blot analyses of F-NIRVS. When probes were obtained by PCRs, the used primers are shown in bold.

AlbFlavi4

**AGGAGCGAAAAGTTCTTGGT**ACTCAGCTCATACTGGCCCGAGTAAACCCGCTCTGGTCTTGCGAGGTCATAATAGTTAAGGAGTGATTCCTTCAAAACTTCAGTGAAGGTTACATCCTCAGGTGGATTTGGCTACGGAAAATCATAGGAGTTGTTTTGAAGAGTACAACTGGTTCGTTATGAATAGGAGAATCTCGAGGGAGGGGCTGCGAGGCCCAACATCTGGTCGCCTGACGACCAGACCGCCTGACCCGGCGGGAAAACGGGTAAAAGCCCTCAAGGCAGGGGGGAAGGTGCTCGCGCCAATAGCCCATGTCAAGAGGGGAGCCCCGGATAGGCAATTGGCCGGTTTATTCGGGGGAAGTGGCCACGAATGGCTGCGCGTTCTAAAAATGGACGTGGGCACAGCTGTTCTGTGGTTGGCGCTCGCAATTGGAGCGTTGGGTCAACGGATGTGGAGCCAATTGGCATCACTAAAACGGCGCGTGAGACGGTTGGAGAAACAACGGAGCCACGGATCCATGATCGGAACATTGACCATGTTCTTTCTCGGATTATGTGTGGCAGAAGTGTTCAGGTACGGCTCGAATGGACAAGGTTCTCTGATATTGGAAAGGGAAAATGGAAGCGATGTGGACAGCTACATTGAGGAACGACTAAGCAGAATGAGCGAGGAAAGGGATAGGAAATC**GTCCGGGTCTGTCGAATCA**

AlbFlavi18_20_28

TGACACTCTTAATATCTTCTCGAATGTTTGTGAAAAGCCTCATTTCCCATGCGAGGACGCCACATACCACACTTGATCCTATGATCACCCACTTCACTGTCTAGGTAGCTCCGGTTACCCGCAGCTTTGTACAGGACAGGGTACACTATGAACATGGCTAAGAAAATGATCACGCAGAATCCGAGTGGAACACCCAAATAGTGCAAGACACTTGGTGATGTGAGAATAACCCCGCCCCCAAACGAGTCCGAGGATGGCATTGGCTTGTATGCGTACTTCGCCGAGCGAGCATAGGATAAGACGCAACTGAGGAGCCAGAGCGTCATGATGAGTAACATGGCAATCGCAAGCCCGAGCGTGAAGGCCATCCCTGATGTGAGCGACCGCTCAGTGAGGATTTGGTCCCGCAGGTTCCCACTCACTATGTCGTGTAGGTCCCACAAGGATGATGCTGTGCTCTTCCAGATGTTGTTCCAATCTATGCTCATCAGTATGTCATAGAGACTGTGACTGCTCCTACAATTCAGGTAGAAGTCGATTGAGAGTTTACGAGTATCCCATTCAAGGTTTTCGAAGCAGTCATCGACGTACTGGGGTTTGTACTCCATCTTGCCATACCGGCTGTACAGCGTGTTTCCGGTGCAAACCCCCTGGTACAATGCTGAATGTCGGCGGTCGCCAGCTTCAGCCCAGTGCCAAGCAAGCCAAATGGGGATGTCATCTCGATCCAGTAACTTAACGAACCTGAGTAGATCCTCTCCTACTAGGGTATACCGTCCTGGTGGCTGGCTGTAAGCGGCTTCCTCCTGCATAAAGGTCATGCCTATCTGGTCCAAGATCATCTGTGCTTCGGGCCAACATGCCCACGACACGGGGTTCTCCTCCGTTTCACTGTCCACTGGATAAACATAACACCCTGGGGCACGGCGCCCTGTGCGCCCTCTTCGCTGGATCATGGACGGCGTTGTGATGTTGGTTTCAGCGAGTTTGACCCTTCTCTCCGAGTCAACCACTGGCCTCAAGACTCTCCTAGTGTCAATCACCGTGTCAACACCAAGATTTGCTCCCATCTCAG

# List of primers used for qPCR-based copy number. The respective amplicon size for each primer set is shown in brackets.

AlbFlavi19, AlbFlavi33 (88 bp)

19_33F2: TCAACGTGGCCTGTTCCA

19_33R2: CTGTGATTGGAGCAACTGTGA

AlbFlavi24, AlbFlavi33 (80 bp)

24_33F1: AATAGCCAAGCACAGGAGGT

24_33R1: GATCGGATTTTCACCCAGCG

AlbFlavi18, AlbFlavi20, AlbFlavi28 (100 bp)

18-20-28F1: GGCTCCTCAGTTGCGTCTTA

18-20-28R1: GATGTGAGAATAACCCCGCC

AlbFlavi26, AlbFlavi37, AlbFlavi42 (86 bp)

26-37-42F2: ATCTTTTCCTCTGTTGGGCTT

26-37-42R2: CACATGTTCAGCAGCACGTC

AlbFlavi22, AlbFlavi23, AlbFlav27, AlbFlavi26, AlbFlavi37 (80 bp)

22-23-27-26-37F1: TTCCGCCCGAAGTTTTGGTA

22-23-27-26-37R1: TATCAGTGATGCAACGATGGC

AlbFlavi22, AlbFlavi25, AlbFlav27, AlbFlavi26, AlbFlavi37 (91 bp)

22-25-27-26-37F1: CGTGTGGTTTTGGACTGTGT

22-25-27-26-37R1: TAGTAGTACTGCTGCCACCG

AlbFlavi31, AlbFlavi32, AlbFlavi34 (95 bp)

31-32-34F1: GTTGCCTTACTGATGCTCGG

31-32-34R1: GCGTTCTTGCCAGAGTTCC

AlbFlavi7 (130 bp)

AlbFlavi7F3: GATTGTTTCCCATGGCCACA

AlbFlavi7R3: GTTTGAGGACCCTGAACATCC

AlbFlavi8, AlbFlavi41 (141 bp)

AlbFlavi8F1: GTCAGTCTCCTTCTCGGCAT

AlbFlavi8R1: GAAACTCGAAAACGCACCCT

AlbFlavi3 (118 bp)

AlbFlavi3F4: GGGGCATCTGTTGTACCTCA

AlbFlavi3R4: CCTTGTGGGCGTGTCTATTG

AlbFlavi36 (166 bp)

AlbFlavi36F1: CAAGTACAAGTGGGCCCCTA

AlbFlavi36R1: ATTGTAGCCGCGAGTTAGGA

AlbFlavi4 (131 bp)

AlbFlavi4F1: AACGGATGTGGAGCCAATTG

AlbFlavi4R1: CCTGAACACTTCTGCCACAC

AlbFlavi6 (191 bp)

AlbFlavi6F1: ACTCTCCAACATACGGGCAA

AlbFlavi6R1: TATCCGCCACCATGAGACTC

AlbFlavi2 (146 bp)

AlbFlavi2F2: ACCCCATCTTGCCCATATCC

AlbFlavi2R2: TGGAAAAGCTCTGTCATGCG

AlbFlavi10 (109 bp)

AlbFlavi10F1: GAAATTCTCACGGCTGACCC

AlbFlavi10R1: GGCAAAGGGATCTCGGACTA

AlbFlavi1 (117 bp)^a^

AlbFlavi1F1: GGGTGAGTGGAATGGAGGAA

AlbFlavi1R1: GTTCCGCTATCGTTCCACAC

AlbFlavi12_17 (171 bp)

AlbFlavi 12_17 F1: CACTCGGTTGCGTCCTATTG

AlbFlavi 12_17 R1: GCAGCTCTTCCTCATCTTGC

AlbFlavi 1, 12_17 (145 bp)^a^

AlbFlavi 1_12 F: GAAACGGTAGATGCTTGGCC

AlbFlavi 1_12 R: CACACCCGCAACATGTCATC

^a^we noticed that upstream of AlbFlavi12_17 on supercontig JXUM01S004920 and downstream of AlbFlavi1 on supercontig JXUM01S000094, there are two shorter sequences that match to portions of AlbFlavi1 and AlbFlavi12_17, thus possibly increasing the expected number by qPCR to 3 or 4 copies.

# List of primers used for tracing N-Gs expression profiles. The respective amplicon size for each primer set is shown in brackets.

AALF005432 (86 bp)

AlbFv34-alt-F: CTTGCGACCCATGGTCTTCT

AlbFv34-alt-R: GTCCTCGGCGCTGAATCATA

AALF025780 (83 bp)

AR12_qF: CCAGAATTACGCCCGGTTTC

AR12_qR: GGCCTTCCTTCACCATCCAT

AALF025780/AALF000476 (101 bp)

AR12-15_qF: TCGAAAGCTCAACCACCAAA

AR12-15_qR: AGTTCTTCACGAGCTCTCCA

AALF000477 (100 bp)

AR18-28_qF: CATCGGACGAGCAGGTGA

AR18-28_qR: CTGCTTTCCAACAGGACGT

AALF025779 (112 bp)

AR9-28_qF: CGATGTTCCACGGCATACAG

AR9-28_qR: AACGACCGGAAATGCAGAAG

AALF020122 (117 bp)

AR52_qF: GGAGTTCTTCGTTGTCACCG

AR52_qR: TCGGAGGCAGCTAACATCTT

AALF004130 (100 bp)

AR85_qF: CACGACGAATGACCAACTCC

AR85_qR: CGCATTTATCGGAGTGTCGG

Nucleosome assembly protein (nap) (115 bp)

napF: TTACGTTCTTGCTCGGGTTCCACT

napR: TAAGACGTACTTCATGCGCTGCCA

# List of primers used for population genetics. The respective amplicon size for each primer set is shown in brackets.

AlbFlavi12_17 (436 bp)

5617-91F: TTTCTACTGCCTCGCCATGA

5617CD-R: GACGCATCCTAATTGTTCCGA

AlbFlavi1, AlbFlavi12_17 (233 bp-AlbFlavi1; 1262 bp-AlbFlavi12_17)

5617-91F: TTTCTACTGCCTCGCCATGA

5617-91R: GAGTTGAATGGAGGAAGTCGTG

AlbFlavi10 (1583 bp)

5171Fext: CACCCACATCCGAAAGCTTC

5171Rex: TTCCCGCGACCAGTATTCTT

AlbFlavi2 (960 bp)

157AF: TCACAAACGCATGCTACACC

157AR: TTCATTTGAGAGCAAGCGGG

AlbFlavi36 (1055 bp)

14636EF: AAGTTCGTGTTTTGGGTGCA

14636ER: GATGCGCTCTCCTACTCACT

AlbFlavi4 (690 bp)

1256F: AGGAGCGAAAAGTTCTTGGT

1256R: TGATTCGACAGACCCGGAC

AlbFlavi8, AlbFlavi41 (681 bp)

4896-8815F: CCGTGACGCTTGATGAGTTT

4896-8815Rext: TGGTACTATCAACGGCATCTCT

AlbRha1 (1090 bp)

AR1_Fext: GGAGTTGCTGCCTCGGTC

AR1_Rext: GCATTTCCTGGGCTCCTAAGT

AlbRha7 (862 bp)

AR7_Fext: CGAGAGAAGGTGGACTGGTT

AR7_Rext: ACAGTTCGTCACGCCACTTA

AlbRha14 (350 bp)

AR14_Fext: TAACTGTTCGCTAGTGGACTCG

AR14_Rext: GCTTCAAACATTGCGCGTGA

AlbRha36 (829 bp)

AR36_Fext: CAACAACCGCGAGAAGAAGC

AR36_Rext: AATACCATTCCAGGGCGTCC

AlbRha52 (968 bp)

AR52_F4: GAGAAGCCAATGACCCTGTGT

AR52_Rext: GATTGACTGATGGACCAAGAACA

AlbRha85 (638 bp)

AR85_F2: GACCCCTCTGTCCTGGATCA

AR85_Rext: TCGAGCCCCATATTTTGAAGC

# Primers used to confirm data from soft-clipped reads

AlbFlavi6-AlbFlavi7 contiguity

AF6_F: TAAGCGAGTCTCATGGTGGC

AF7_Rext: GAACCTAGTAACGCCGCGAG

AlbFlavi10

5171Fext: CACCCACATCCGAAAGCTTC

5171Rex: TTCCCGCGACCAGTATTCTT

# DAS genetic distance

DAS genetic distance across five geographic populations using data from 1) all tested NIRVS; 2) F-NIRVS; 3) R-NIRVS; 4) NIRVS in intergenic regions of the genome or 5) NIRVS in piRNA clusters.

1) All tested NIRVS

China 0 0.2 0.240 0.23 0.205

La Reunion 0.2 0 0.337 0.306 0.214

Italy 0.240 0.337 0 0.004 0.185

Thailand 0.23 0.306 0.004 0 0.151

USA 0.205 0.214 0.185 0.151 0

2) F-NIRVS

China 0 0.125 0.358 0.356 0.357

La Reunion 0.125 0 0.293 0.348 0.208

Italy 0.358 0.293 0 0.0161 0.209

Thailand 0.356 0.348 0.016 0 0.222

USA 0.359 0.208 0.209 0.222 0

3) R-NIRVS

China 0 0.325 0.082 0 0

La Reunion 0.325 0 0.389 0.229 0.222

Italy 0.082 0.389 0 0 0.156

Thailand 0 0.226 0 0 0.018

USA 0 0.223 0.156 0.017 0

4) NIRVS in intergenic regions

China 0 0.174 0.486 0.413 0.342

La Reunion 0.174 0 0.460 0.402 0.2

Italy 0.486 0.460 0 0.068 0.313

Thailand 0.413 0.402 0.068 0 0.2

USA 0.342 0.2 0.313 0.2 0

5) NIRVS in piRNA clusters

China 0 0.324 0 0.004 0.110

La Reunion 0.324 0 0.217 0.2 0.342

Italy 0 0.217 0 0 0.092

Thailand 0.004 0.2 0 0 0.151

USA 0.110 0.342 0.09 0.151 0

Supplementary Tables

# Gene evolutionary rate.

Due to its size, this table is reported as ‘Supplementary_Table1.xlsx’ file.

# NIRVS loci detection in SSM.

For each of the 16 SSM ‘1’ value indicates the detection of a F- or R-NIRVS. Otherwise ‘0’ value is reported. The detection frequency for each locus across the 16 SSM is also reported.

| **F-NIRVS** | **Detection Frequency** | **SSM 1** | **SSM 2** | **SSM 3** | **SSM 4** | **SSM 5** | **SSM 6** | **SSM 7** | **SSM 8** | **SSM 9** | **SSM 10** | **SSM 11** | **SSM 12** | **SSM 13** | **SSM 14** | **SSM 15** | **SSM 16** |
| --- | --- | --- | --- | --- | --- | --- | --- | --- | --- | --- | --- | --- | --- | --- | --- | --- | --- |
| AlbFlavi1 | 0,9375 | 1 | 1 | 1 | 0 | 1 | 1 | 1 | 1 | 1 | 1 | 1 | 1 | 1 | 1 | 1 | 1 |
| AlbFlavi10 | 0,8125 | 1 | 0 | 1 | 1 | 1 | 1 | 1 | 0 | 0 | 1 | 1 | 1 | 1 | 1 | 1 | 1 |
| AlbFlavi12_17 | 0,875 | 1 | 1 | 1 | 1 | 1 | 1 | 0 | 1 | 1 | 1 | 0 | 1 | 1 | 1 | 1 | 1 |
| AlbFlavi18 | 0,25 | 0 | 0 | 0 | 0 | 0 | 0 | 0 | 0 | 0 | 1 | 0 | 0 | 0 | 1 | 1 | 1 |
| AlbFlavi19 | 0 | 0 | 0 | 0 | 0 | 0 | 0 | 0 | 0 | 0 | 0 | 0 | 0 | 0 | 0 | 0 | 0 |
| AlbFlavi2 | 0,9375 | 1 | 1 | 1 | 1 | 1 | 1 | 1 | 1 | 1 | 1 | 1 | 1 | 1 | 1 | 0 | 1 |
| AlbFlavi20 | 0,125 | 0 | 0 | 0 | 0 | 0 | 0 | 0 | 0 | 0 | 0 | 0 | 0 | 0 | 1 | 0 | 1 |
| AlbFlavi22 | 0,9375 | 1 | 1 | 1 | 1 | 1 | 0 | 1 | 1 | 1 | 1 | 1 | 1 | 1 | 1 | 1 | 1 |
| AlbFlavi23 | 0,8125 | 1 | 1 | 0 | 1 | 0 | 0 | 1 | 1 | 1 | 1 | 1 | 1 | 1 | 1 | 1 | 1 |
| AlbFlavi24 | 0,75 | 1 | 0 | 1 | 1 | 0 | 1 | 1 | 0 | 0 | 1 | 1 | 1 | 1 | 1 | 1 | 1 |
| AlbFlavi25 | 1 | 1 | 1 | 1 | 1 | 1 | 1 | 1 | 1 | 1 | 1 | 1 | 1 | 1 | 1 | 1 | 1 |
| AlbFlavi26 | 0,9375 | 1 | 1 | 1 | 1 | 1 | 1 | 1 | 1 | 0 | 1 | 1 | 1 | 1 | 1 | 1 | 1 |
| AlbFlavi27 | 0,875 | 1 | 1 | 1 | 0 | 1 | 1 | 1 | 1 | 0 | 1 | 1 | 1 | 1 | 1 | 1 | 1 |
| AlbFlavi28 | 0,6875 | 1 | 0 | 1 | 0 | 1 | 1 | 1 | 0 | 0 | 1 | 0 | 1 | 1 | 1 | 1 | 1 |
| AlbFlavi3 | 0,9375 | 1 | 1 | 1 | 1 | 1 | 1 | 1 | 1 | 1 | 1 | 1 | 1 | 1 | 1 | 0 | 1 |
| AlbFlavi31 | 0 | 0 | 0 | 0 | 0 | 0 | 0 | 0 | 0 | 0 | 0 | 0 | 0 | 0 | 0 | 0 | 0 |
| AlbFlavi32 | 0 | 0 | 0 | 0 | 0 | 0 | 0 | 0 | 0 | 0 | 0 | 0 | 0 | 0 | 0 | 0 | 0 |
| AlbFlavi33 | 0 | 0 | 0 | 0 | 0 | 0 | 0 | 0 | 0 | 0 | 0 | 0 | 0 | 0 | 0 | 0 | 0 |
| AlbFlavi34 | 1 | 1 | 1 | 1 | 1 | 1 | 1 | 1 | 1 | 1 | 1 | 1 | 1 | 1 | 1 | 1 | 1 |
| AlbFlavi36 | 0,8125 | 1 | 0 | 1 | 1 | 1 | 1 | 1 | 0 | 0 | 1 | 1 | 1 | 1 | 1 | 1 | 1 |
| AlbFlavi37 | 0,875 | 1 | 1 | 1 | 1 | 0 | 0 | 1 | 1 | 1 | 1 | 1 | 1 | 1 | 1 | 1 | 1 |
| AlbFlavi38 | 0 | 0 | 0 | 0 | 0 | 0 | 0 | 0 | 0 | 0 | 0 | 0 | 0 | 0 | 0 | 0 | 0 |
| AlbFlavi39 | 0 | 0 | 0 | 0 | 0 | 0 | 0 | 0 | 0 | 0 | 0 | 0 | 0 | 0 | 0 | 0 | 0 |
| AlbFlavi4 | 0,875 | 1 | 1 | 1 | 1 | 1 | 1 | 0 | 1 | 1 | 1 | 1 | 0 | 1 | 1 | 1 | 1 |
| AlbFlavi40 | 0 | 0 | 0 | 0 | 0 | 0 | 0 | 0 | 0 | 0 | 0 | 0 | 0 | 0 | 0 | 0 | 0 |
| AlbFlavi42 | 0,6875 | 0 | 1 | 0 | 1 | 0 | 0 | 1 | 1 | 1 | 1 | 1 | 0 | 1 | 1 | 1 | 1 |
| AlbFlavi6 | 1 | 1 | 1 | 1 | 1 | 1 | 1 | 1 | 1 | 1 | 1 | 1 | 1 | 1 | 1 | 1 | 1 |
| AlbFlavi7 | 1 | 1 | 1 | 1 | 1 | 1 | 1 | 1 | 1 | 1 | 1 | 1 | 1 | 1 | 1 | 1 | 1 |
| AlbFlavi8-41 | 0,25 | 0 | 0 | 0 | 0 | 1 | 0 | 1 | 0 | 0 | 0 | 0 | 0 | 0 | 1 | 1 | 0 |

| **R-NIRVS** | **Detection Frequency** | **SSM 1** | **SSM 2** | **SSM 3** | **SSM 4** | **SSM 5** | **SSM 6** | **SSM 7** | **SSM 8** | **SSM 9** | **SSM 10** | **SSM 11** | **SSM 12** | **SSM 13** | **SSM 14** | **SSM 15** | **SSM 16** |
| --- | --- | --- | --- | --- | --- | --- | --- | --- | --- | --- | --- | --- | --- | --- | --- | --- | --- |
| AlbRha1 | 1 | 1 | 1 | 1 | 1 | 1 | 1 | 1 | 1 | 1 | 1 | 1 | 1 | 1 | 1 | 1 | 1 |
| AlbRha10 | 0,25 | 0 | 1 | 0 | 0 | 0 | 1 | 0 | 1 | 1 | 0 | 0 | 0 | 0 | 0 | 0 | 0 |
| AlbRha11 | 0,375 | 0 | 1 | 1 | 1 | 0 | 0 | 0 | 1 | 0 | 0 | 0 | 1 | 0 | 0 | 1 | 0 |
| AlbRha12 | 1 | 1 | 1 | 1 | 1 | 1 | 1 | 1 | 1 | 1 | 1 | 1 | 1 | 1 | 1 | 1 | 1 |
| AlbRha14 | 1 | 1 | 1 | 1 | 1 | 1 | 1 | 1 | 1 | 1 | 1 | 1 | 1 | 1 | 1 | 1 | 1 |
| AlbRha15 | 1 | 1 | 1 | 1 | 1 | 1 | 1 | 1 | 1 | 1 | 1 | 1 | 1 | 1 | 1 | 1 | 1 |
| AlbRha18 | 0,9375 | 1 | 1 | 1 | 1 | 1 | 1 | 1 | 1 | 1 | 1 | 1 | 0 | 1 | 1 | 1 | 1 |
| AlbRha2 | 0,5 | 1 | 0 | 1 | 0 | 1 | 0 | 1 | 0 | 1 | 0 | 1 | 0 | 1 | 0 | 0 | 1 |
| AlbRha28 | 1 | 1 | 1 | 1 | 1 | 1 | 1 | 1 | 1 | 1 | 1 | 1 | 1 | 1 | 1 | 1 | 1 |
| AlbRha3 | 0,875 | 1 | 1 | 1 | 1 | 0 | 1 | 1 | 1 | 1 | 1 | 1 | 1 | 0 | 1 | 1 | 1 |
| AlbRha32 | 0,25 | 0 | 0 | 0 | 1 | 0 | 0 | 0 | 0 | 0 | 0 | 1 | 0 | 1 | 0 | 0 | 1 |
| AlbRha33 | 1 | 1 | 1 | 1 | 1 | 1 | 1 | 1 | 1 | 1 | 1 | 1 | 1 | 1 | 1 | 1 | 1 |
| AlbRha36 | 1 | 1 | 1 | 1 | 1 | 1 | 1 | 1 | 1 | 1 | 1 | 1 | 1 | 1 | 1 | 1 | 1 |
| AlbRha38 | 0,6875 | 0 | 1 | 0 | 1 | 1 | 1 | 0 | 1 | 1 | 1 | 0 | 1 | 1 | 0 | 1 | 1 |
| AlbRha4 | 0,75 | 1 | 1 | 1 | 1 | 0 | 0 | 1 | 1 | 0 | 1 | 1 | 1 | 0 | 1 | 1 | 1 |
| AlbRha41 | 0,25 | 0 | 0 | 0 | 0 | 0 | 0 | 1 | 0 | 0 | 0 | 1 | 0 | 0 | 1 | 0 | 1 |
| AlbRha42 | 0,25 | 0 | 0 | 0 | 0 | 0 | 0 | 1 | 0 | 0 | 0 | 1 | 0 | 0 | 1 | 0 | 1 |
| AlbRha43 | 0 | 0 | 0 | 0 | 0 | 0 | 0 | 0 | 0 | 0 | 0 | 0 | 0 | 0 | 0 | 0 | 0 |
| AlbRha44 | 1 | 1 | 1 | 1 | 1 | 1 | 1 | 1 | 1 | 1 | 1 | 1 | 1 | 1 | 1 | 1 | 1 |
| AlbRha45 | 0,3125 | 0 | 0 | 0 | 0 | 1 | 1 | 1 | 0 | 0 | 0 | 0 | 1 | 0 | 0 | 1 | 0 |
| AlbRha48 | 0,875 | 1 | 1 | 1 | 1 | 1 | 1 | 1 | 1 | 1 | 1 | 0 | 1 | 1 | 1 | 1 | 0 |
| AlbRha49 | 1 | 1 | 1 | 1 | 1 | 1 | 1 | 1 | 1 | 1 | 1 | 1 | 1 | 1 | 1 | 1 | 1 |
| AlbRha52 | 1 | 1 | 1 | 1 | 1 | 1 | 1 | 1 | 1 | 1 | 1 | 1 | 1 | 1 | 1 | 1 | 1 |
| AlbRha58 | 0,875 | 1 | 1 | 1 | 1 | 0 | 0 | 1 | 1 | 1 | 1 | 1 | 1 | 1 | 1 | 1 | 1 |
| AlbRha62 | 0,875 | 1 | 1 | 1 | 1 | 0 | 0 | 1 | 1 | 1 | 1 | 1 | 1 | 1 | 1 | 1 | 1 |
| AlbRha66 | 0,4375 | 1 | 0 | 1 | 0 | 1 | 1 | 0 | 0 | 0 | 1 | 0 | 1 | 0 | 0 | 0 | 1 |
| AlbRha7 | 1 | 1 | 1 | 1 | 1 | 1 | 1 | 1 | 1 | 1 | 1 | 1 | 1 | 1 | 1 | 1 | 1 |
| AlbRha71 | 0,625 | 1 | 0 | 1 | 0 | 1 | 1 | 1 | 0 | 0 | 0 | 1 | 1 | 1 | 1 | 1 | 0 |
| AlbRha73 | 0,5 | 0 | 0 | 0 | 1 | 0 | 0 | 1 | 1 | 1 | 0 | 0 | 0 | 1 | 1 | 1 | 1 |
| AlbRha74 | 0,5 | 0 | 0 | 0 | 1 | 0 | 0 | 1 | 1 | 1 | 0 | 0 | 0 | 1 | 1 | 1 | 1 |
| AlbRha79 | 0 | 0 | 0 | 0 | 0 | 0 | 0 | 0 | 0 | 0 | 0 | 0 | 0 | 0 | 0 | 0 | 0 |
| AlbRha80 | 0 | 0 | 0 | 0 | 0 | 0 | 0 | 0 | 0 | 0 | 0 | 0 | 0 | 0 | 0 | 0 | 0 |
| AlbRha83 | 1 | 1 | 1 | 1 | 1 | 1 | 1 | 1 | 1 | 1 | 1 | 1 | 1 | 1 | 1 | 1 | 1 |
| AlbRha84 | 1 | 1 | 1 | 1 | 1 | 1 | 1 | 1 | 1 | 1 | 1 | 1 | 1 | 1 | 1 | 1 | 1 |
| AlbRha85 | 1 | 1 | 1 | 1 | 1 | 1 | 1 | 1 | 1 | 1 | 1 | 1 | 1 | 1 | 1 | 1 | 1 |
| AlbRha87 | 0,125 | 0 | 0 | 1 | 0 | 0 | 0 | 0 | 0 | 0 | 0 | 1 | 0 | 0 | 0 | 0 | 0 |
| AlbRha88 | 0,75 | 1 | 1 | 1 | 0 | 1 | 1 | 1 | 1 | 1 | 0 | 1 | 1 | 1 | 0 | 0 | 1 |
| AlbRha9 | 1 | 1 | 1 | 1 | 1 | 1 | 1 | 1 | 1 | 1 | 1 | 1 | 1 | 1 | 1 | 1 | 1 |
| AlbRha92 | 0,875 | 1 | 0 | 1 | 1 | 1 | 1 | 1 | 0 | 1 | 1 | 1 | 1 | 1 | 1 | 1 | 1 |
| AlbRha94 | 0,8125 | 1 | 1 | 1 | 1 | 1 | 1 | 1 | 1 | 1 | 0 | 1 | 1 | 1 | 0 | 1 | 0 |
| AlbRha95 | 0 | 0 | 0 | 0 | 0 | 0 | 0 | 0 | 0 | 0 | 0 | 0 | 0 | 0 | 0 | 0 | 0 |
| AlbRha96 | 1 | 1 | 1 | 1 | 1 | 1 | 1 | 1 | 1 | 1 | 1 | 1 | 1 | 1 | 1 | 1 | 1 |

# Kolmogorov-Smirnov test results.

The distribution of LoP of each locus and the distribution of conserved gene were tested with the Kolmogorov-Smirnov test. The threshold of significance was adjusted with the Bonferroni correction and loci were separated according to the adjusted significance of the test (-log10 0.00048 = 3.32). Results with Fold Change (FC) different from 0 are summarized in Volcano Plot. Significantly/not significantly results different compared to the conserved genes are shown in the following two table.

| **Class** | **Group** | **Locus name** | **Fold Change** | **log2 Fold Change** | **P value** | **-log10 P value** |
| --- | --- | --- | --- | --- | --- | --- |
| F-NIRVS | coding sequence | AlbFlavi24 | 0 | -Inf | 2.21305081804513e-06 | 5.65500861330131 |
| F-NIRVS | coding sequence | AlbFlavi4 | 0 | -Inf | 5.11648286383171e-06 | 5.29102847607729 |
| F-NIRVS | intergenic | AlbFlavi28 | 0 | -Inf | 4.18564942502586e-05 | 4.37823714942128 |
| F-NIRVS | intergenic | AlbFlavi42 | 0 | -Inf | 0.000324207911207353 | 3.48917639185709 |
| F-NIRVS | intergenic | AlbFlavi10 | 0.333793423076834 | -1.58297256615424 | 1.17801421506414e-06 | 5.9288494688981 |
| F-NIRVS | intergenic | AlbFlavi36 | 0.456803667281733 | -1.13035386198886 | 1.17801421506414e-06 | 5.9288494688981 |
| F-NIRVS | PIRC | AlbFlavi12_17 | 4.1074354792889 | 2.03823791310815 | 3.43715493050789e-05 | 4.46380089150311 |
|  |  |  |  |  |  |  |
| Gene-NIRVS | gene | AALF007713 | 0 | -Inf | 2.25070349468837e-07 | 6.64768171472955 |
| Gene-NIRVS | gene | AALF023281 | 0 | -Inf | 2.21305081804513e-06 | 5.65500861330131 |
| Gene-NIRVS | gene | AALF000478 | 0.070080892418664 | -3.83483504326339 | 2.25070349468837e-07 | 6.64768171472955 |
| Gene-NIRVS | gene | AALF004130 | 0.319885363781966 | -1.64437311081838 | 1.56229788161877e-06 | 5.80623615611221 |
| Gene-NIRVS | gene | AALF025779 | 0.493180276665625 | -1.01981299045413 | 0.000246819608172966 | 3.60762034146598 |
| Gene-NIRVS | gene | AALF020122 | 3.10958900864474 | 1.63672391335518 | 9.57023478420638e-06 | 5.0190774076532 |
|  |  |  |  |  |  |  |
| Gene-RNAi | gene | AALF005498 | 0 | -Inf | 2.25070349468837e-07 | 6.64768171472955 |
| Gene-RNAi | gene | AALF006056 | 0.0866538178240608 | -3.52859287622884 | 2.25070349468837e-07 | 6.64768171472955 |
| Gene-RNAi | gene | AALF005499 | 0.220819886519031 | -2.17905799120216 | 2.25070349468837e-07 | 6.64768171472955 |
| Gene-RNAi | gene | AALF016369 | 2.89509492518397 | 1.53361065247945 | 5.17362004452959e-05 | 4.28620546943922 |
| Gene-RNAi | gene | AALF020776 | 2.94952521242079 | 1.56048274131738 | 9.57023478420638e-06 | 5.0190774076532 |
|  |  |  |  |  |  |  |
| Gene-Fast evolving | gene | AALF019413 | 0 | -Inf | 3.77111933680396e-07 | 6.42352972415884 |
| Gene-Fast evolving | gene | AALF004733 | 0 | -Inf | 1.56229788161877e-06 | 5.80623615611221 |
| Gene-Fast evolving | gene | AALF028390 | 2.69424898083801 | 1.42988317924209 | 2.25070349468837e-07 | 6.64768171472955 |
| Gene-Fast evolving | gene | AALF009839 | 2.94388010059554 | 1.55771891399047 | 2.25070349468837e-07 | 6.64768171472955 |
| Gene-Fast evolving | gene | AALF024551 | 4.08548440015461 | 2.03050714330847 | 5.17362004452959e-05 | 4.28620546943922 |
| Gene-Fast evolving | gene | AALF018679 | 4.22091574147227 | 2.07755603029693 | 2.25070349468837e-07 | 6.64768171472955 |
| Gene-Fast evolving | gene | AALF010748 | 5.86951136080968 | 2.55324040338987 | 2.25070349468837e-07 | 6.64768171472955 |
| Gene-Fast evolving | gene | AALF017064 | 7.14320282682611 | 2.83657108634436 | 2.25070349468837e-07 | 6.64768171472955 |
| Gene-Fast evolving | gene | AALF026991 | 8.46649209608714 | 3.08176434454485 | 2.25070349468837e-07 | 6.64768171472955 |
|  |  |  |  |  |  |  |
| R-NIRVS | PIRC | AlbRha14 | 0 | -Inf | 2.25070349468837e-07 | 6.64768171472955 |
| R-NIRVS | intergenic | AlbRha44 | 0 | -Inf | 2.25070349468837e-07 | 6.64768171472955 |
| R-NIRVS | coding sequence | AlbRha92 | 0 | -Inf | 6.53981905851531e-07 | 6.18443426742121 |
| R-NIRVS | coding sequence | AlbRha9 | 0 | -Inf | 1.56229788161877e-06 | 5.80623615611221 |
| R-NIRVS | intergenic | AlbRha2 | 0 | -Inf | 4.66182022859085e-05 | 4.33144447797038 |
| R-NIRVS | coding sequence | AlbRha28 | 0.0695890966823927 | -3.84499490948361 | 2.25070349468837e-07 | 6.64768171472955 |
| R-NIRVS | coding sequence | AlbRha85 | 0.198577146978542 | -2.33222849335948 | 2.25070349468837e-07 | 6.64768171472955 |
| R-NIRVS | intergenic | AlbRha3 | 2.97789696472923 | 1.57429383751781 | 2.73158538934837e-05 | 4.56358521889343 |
| R-NIRVS | intergenic | AlbRha96 | 3.00358785697045 | 1.58668686428449 | 5.17362004452959e-05 | 4.28620546943922 |
| R-NIRVS | coding sequence | AlbRha52 | 3.10842776918953 | 1.63618505523283 | 9.57023478420638e-06 | 5.0190774076532 |
| R-NIRVS | intergenic | AlbRha83 | 3.85416535471713 | 1.94641846870497 | 1.56229788161877e-06 | 5.80623615611221 |
| R-NIRVS | intergenic | AlbRha1 | 4.23083803128729 | 2.08094345621392 | 9.57023478420638e-06 | 5.0190774076532 |
| R-NIRVS | intergenic | AlbRha84 | 4.71090084429499 | 2.23600296627682 | 2.25070349468837e-07 | 6.64768171472955 |
|  |  |  |  |  |  |  |
| **Class** | **Group** | **Locus name** | **Fold Change** | **log2 Fold Change** | **P value** | **-log10 P value** |
| F-NIRVS | intergenic | AlbFlavi26 | 0 | -Inf | 0.000483850520987206 | 3.31528878698603 |
| F-NIRVS | PIRC | AlbFlavi18 | 0 | -Inf | 0.00332311453110412 | 2.47845469050902 |
| F-NIRVS | intergenic | AlbFlavi8_41 | 0 | -Inf | 0.0546463301138636 | 1.26243899866972 |
| F-NIRVS | PIRC | AlbFlavi20 | 0 | -Inf | 0.0571296698968321 | 1.24313828534604 |
| F-NIRVS | PIRC | AlbFlavi2 | 0.170973211676568 | -2.54815779555908 | 0.00758906849936414 | 2.11981152718917 |
| F-NIRVS | intergenic | AlbFlavi27 | 0.408925619680039 | -1.29008964261863 | 0.000982018472579393 | 3.00788034269794 |
| F-NIRVS | intergenic | AlbFlavi1 | 0.574866450854548 | -0.798701257209673 | 0.0244492504105508 | 1.61173445136866 |
| F-NIRVS | PIRC | AlbFlavi3 | 0.665254257592685 | -0.588022256437016 | 0.00702180772685168 | 2.15355106668362 |
| F-NIRVS | intergenic | AlbFlavi37 | 0.69215126537196 | -0.53084073050244 | 0.03171693775341 | 1.49870875013739 |
| F-NIRVS | intergenic | AlbFlavi25 | 0.698278564493426 | -0.518125408823792 | 0.0366310527071189 | 1.43615060035475 |
| F-NIRVS | intergenic | AlbFlavi23 | 0.773966538711489 | -0.36965689993695 | 0.239167984323949 | 0.621296956631917 |
| F-NIRVS | PIRC | AlbFlavi22 | 0.862299676281822 | -0.213738756488517 | 0.167819849961045 | 0.775156671524649 |
| F-NIRVS | coding sequence | AlbFlavi34 | 1.46974368443031 | 0.555564578569319 | 0.0126594276445153 | 1.89758592910917 |
| F-NIRVS | intergenic | AlbFlavi7 | 1.92267700386022 | 0.943116420468773 | 0.0126594276445153 | 1.89758592910917 |
| F-NIRVS | intergenic | AlbFlavi6 | 2.43216716628453 | 1.28224239065713 | 0.00386090824467955 | 2.41331051935571 |
|  |  |  |  |  |  |  |
| Gene-NIRVS | gene | AALF008363 | 0.796583995359737 | -0.32810160093356 | 0.221042627624286 | 0.655523965424403 |
| Gene-NIRVS | gene | AALF000477 | 0.834337285376978 | -0.261297375999029 | 0.159348673362578 | 0.797651547839513 |
| Gene-NIRVS | gene | AALF003313 | 0.841720195096215 | -0.24858736306155 | 0.210551632726011 | 0.676641386487136 |
| Gene-NIRVS | gene | AALF000476 | 1.37151941232086 | 0.45577504206285 | 0.210551632726011 | 0.676641386487136 |
| Gene-NIRVS | gene | AALF005432 | 1.57058478515725 | 0.651301826354576 | 0.00386090824467955 | 2.41331051935571 |
| Gene-NIRVS | gene | AALF025780 | 2.05595158890618 | 1.03980629407106 | 0.00386090824467955 | 2.41331051935571 |
|  |  |  |  |  |  |  |
| Gene-RNAi | gene | AALF006534 | 0.526258931148629 | -0.926155282500252 | 0.00386090824467955 | 2.41331051935571 |
| Gene-RNAi | gene | AALF025916 | 0.537003475016998 | -0.896996670785485 | 0.00386090824467955 | 2.41331051935571 |
| Gene-RNAi | gene | AALF015479 | 0.784121268041051 | -0.35085130372021 | 0.210551632726011 | 0.676641386487136 |
| Gene-RNAi | gene | AALF027719 | 0.791975987641194 | -0.336471405789721 | 0.210551632726011 | 0.676641386487136 |
| Gene-RNAi | gene | AALF008582 | 0.81550568253228 | -0.294233165059244 | 0.355999774456201 | 0.44855027717448 |
| Gene-RNAi | gene | AALF006337 | 0.839340268816291 | -0.252672296601243 | 0.0935316745352756 | 1.02904129023578 |
| Gene-RNAi | gene | AALF025919 | 0.954754172741917 | -0.0667987747440349 | 0.415365335523867 | 0.381569750504046 |
| Gene-RNAi | gene | AALF015406 | 1.41514868174687 | 0.500953636908894 | 0.0366310527071189 | 1.43615060035475 |
| Gene-RNAi | gene | AALF006708 | 1.53870932028252 | 0.62172071587954 | 0.0935316745352756 | 1.02904129023578 |
| Gene-RNAi | gene | AALF018531 | 1.69391253134365 | 0.760359380068727 | 0.0126594276445153 | 1.89758592910917 |
| Gene-RNAi | gene | AALF003156 | 1.72969371440115 | 0.790516595182857 | 0.00386090824467955 | 2.41331051935571 |
| Gene-RNAi | gene | AALF007043 | 1.8549928326874 | 0.891413612578523 | 0.00386090824467955 | 2.41331051935571 |
| Gene-RNAi | gene | AALF007445 | 1.99934611917272 | 0.999528247564902 | 0.0126594276445153 | 1.89758592910917 |
|  |  |  |  |  |  |  |
| Gene-Fast evolving | gene | AALF010877 | 0 | -Inf | 0.00205275342962785 | 2.68766321360217 |
| Gene-Fast evolving | gene | AALF022019 | 1.56634468932992 | 0.647401726592611 | 0.0935316745352756 | 1.02904129023578 |
| Gene-Fast evolving | gene | AALF012271 | 1.80697294984129 | 0.853574909400122 | 0.00386090824467955 | 2.41331051935571 |
| Gene-Fast evolving | gene | AALF009493 | 2.98238985781683 | 1.57646885895467 | 0.00386090824467955 | 2.41331051935571 |
| Gene-Fast evolving | gene | AALF014993 | 3.41950955412851 | 1.77378942026742 | 0.000982018472579393 | 3.00788034269794 |
|  |  |  |  |  |  |  |
| R-NIRVS | intergenic | AlbRha11 | 0 | -Inf | 0.000932811545684142 | 3.03020608716066 |
| R-NIRVS | intergenic | AlbRha66 | 0 | -Inf | 0.0015614626153575 | 2.80646840921688 |
| R-NIRVS | intergenic | AlbRha32 | 0 | -Inf | 0.00332311453110412 | 2.47845469050902 |
| R-NIRVS | intergenic | AlbRha41 | 0 | -Inf | 0.00332311453110412 | 2.47845469050902 |
| R-NIRVS | intergenic | AlbRha42 | 0 | -Inf | 0.00332311453110412 | 2.47845469050902 |
| R-NIRVS | intergenic | AlbRha71 | 0 | -Inf | 0.00480728855351709 | 2.31809980892209 |
| R-NIRVS | intergenic | AlbRha45 | 0 | -Inf | 0.0152520136941316 | 1.81667281346373 |
| R-NIRVS | intergenic | AlbRha10 | 0 | -Inf | 0.0546463301138636 | 1.26243899866972 |
| R-NIRVS | intergenic | AlbRha4 | 0.356395738467685 | -1.48844800866808 | 0.0188071094643257 | 1.72567794767968 |
| R-NIRVS | coding sequence | AlbRha18 | 0.730605404923355 | -0.452835668278482 | 0.060783963590555 | 1.21621098393061 |
| R-NIRVS | intergenic | AlbRha38 | 0.831567821990856 | -0.266094161549653 | 0.268986431613944 | 0.570269626405811 |
| R-NIRVS | PIRC | AlbRha36 | 1.01924929616181 | 0.02750696057999 | 0.210551632726011 | 0.676641386487136 |
| R-NIRVS | intergenic | AlbRha74 | 1.07918695772913 | 0.10994481813825 | 0.13892028431882 | 0.857234336519005 |
| R-NIRVS | intergenic | AlbRha58 | 1.12717286559337 | 0.172708787213461 | 0.296756421164588 | 0.527599875082126 |
| R-NIRVS | intergenic | AlbRha94 | 1.15812511267048 | 0.211791116607969 | 0.0941693840217905 | 1.02609027036624 |
| R-NIRVS | coding sequence | AlbRha15 | 1.37151941232086 | 0.45577504206285 | 0.210551632726011 | 0.676641386487136 |
| R-NIRVS | intergenic | AlbRha48 | 1.48176783453026 | 0.56731942186086 | 0.0478253317437048 | 1.32034200877666 |
| R-NIRVS | intergenic | AlbRha49 | 1.61453443732213 | 0.691118213342104 | 0.0935316745352756 | 1.02904129023578 |
| R-NIRVS | intergenic | AlbRha62 | 1.85252293091315 | 0.889491400620139 | 0.0152520136941316 | 1.81667281346373 |
| R-NIRVS | coding sequence | AlbRha12 | 2.05129890580513 | 1.03653772999564 | 0.00386090824467955 | 2.41331051935571 |
| R-NIRVS | intergenic | AlbRha33 | 2.20899045401009 | 1.14338718420606 | 0.0126594276445153 | 1.89758592910917 |
| R-NIRVS | intergenic | AlbRha88 | 2.22217283523607 | 1.15197103042676 | 0.0133959145762501 | 1.8730276305545 |
| R-NIRVS | intergenic | AlbRha7 | 2.74621457159027 | 1.4574443528249 | 0.00103914936416394 | 2.98332202379027 |
| R-NIRVS | intergenic | AlbRha87 | 25.1725174729963 | 4.65377760078586 | 0.0571296698968321 | 1.24313828534604 |
| R-NIRVS | intergenic | AlbRha73 | 4.29761449417992 | 2.10353607553469 | 0.0310075916430483 | 1.50853196406749 |

Supplementary Figures

# NIRVS occurrence: example of AlbFlavi4 and the sequence-similarity group AlbFlavi18-20-28.

AlbFlavi4 was identified as a unique viral sequence in the AaloF1 genome assembly (Palatini et al., 2017). This result was confirmed by a southern-blotting experiment on a pool of 20 Foshan mosquitoes using a probe for AlbFlavi4, which showed only one band **(A)**. Read-coverage analyses of WGS data from SSM11 and SSM12 support the presence and absence of AlbFlavi4, respectively **(B)**. qPCR data on 18 progenies derived from the mating between SSM11 and SSM12 show the absence or occurrence in hemizygosity of AlbFlavi4 in half of the progeny, respectively **(C)**. This result is consistent with the absence of AlbFlavi4 in SSM12 and its presence in hemizygosity in SSM11. AlbFlavi18, AlbFlavi20 and AlbFlavi28 have >90% sequence identity (Palatini et al., 2017). A southern-blot using as probe a sequence that recognizes all three NIRVS showed two distinct bands **(A)**. The different distribution of these three integrations in SSMs explains why only two and not three bands were molecularly-identified. WGS data on the 16 SSMs showed AlbFlavi20 and AlbFlavi18 are rare, they were found at a frequency of 0.13 and 0.25, respectively (Supplementary Table 2). This is in contrast to AlbFlavi28, which was detected at a frequency of 0.69 in SSMs (Supplementary Table 2). Read-coverage analyses of WGS data from SSM11 and SSM12 support the absence of AlbFlavi18 and AlbFlavi20 in both samples, while AlbFlavi28 is absent in SSM11 but present in SSM12 **(B)**. qPCR data on the progeny of the mating between SSM11 and SSM12 show the absence of amplification in half of the progeny, amplification indicating a hemizygote status in the other half of the progeny. This result is consistent with the attribution of presence/absence of AlbFlavi18, AlbFlavi20 e AlbFlavi28 in SSM11 and SSM12 using the converge-criterion of a minimum of 30 consecutive nucleotides with at least 5 reads of depth of coverage **(C)**.

# Phylogenetic analyses of F- and R-NIRVS.

Molecular phylogenetic analysis by Maximum Likelihood method (timetree) for NIRVS with similarities to the L protein of Rhabdovirus **(A)**; the G protein of representative Rhabdoviruses (Palatini et al., 2017) **(B)**; the NS3 protein of Flaviviruses **(C)**; the NS5 protein of Flaviviruses **(D)**. NIRVS are shown with a box, which is red if they belong to the core of NIRVS always identified, otherwise the box is blue.

**(A) Rhabdovirus L protein**


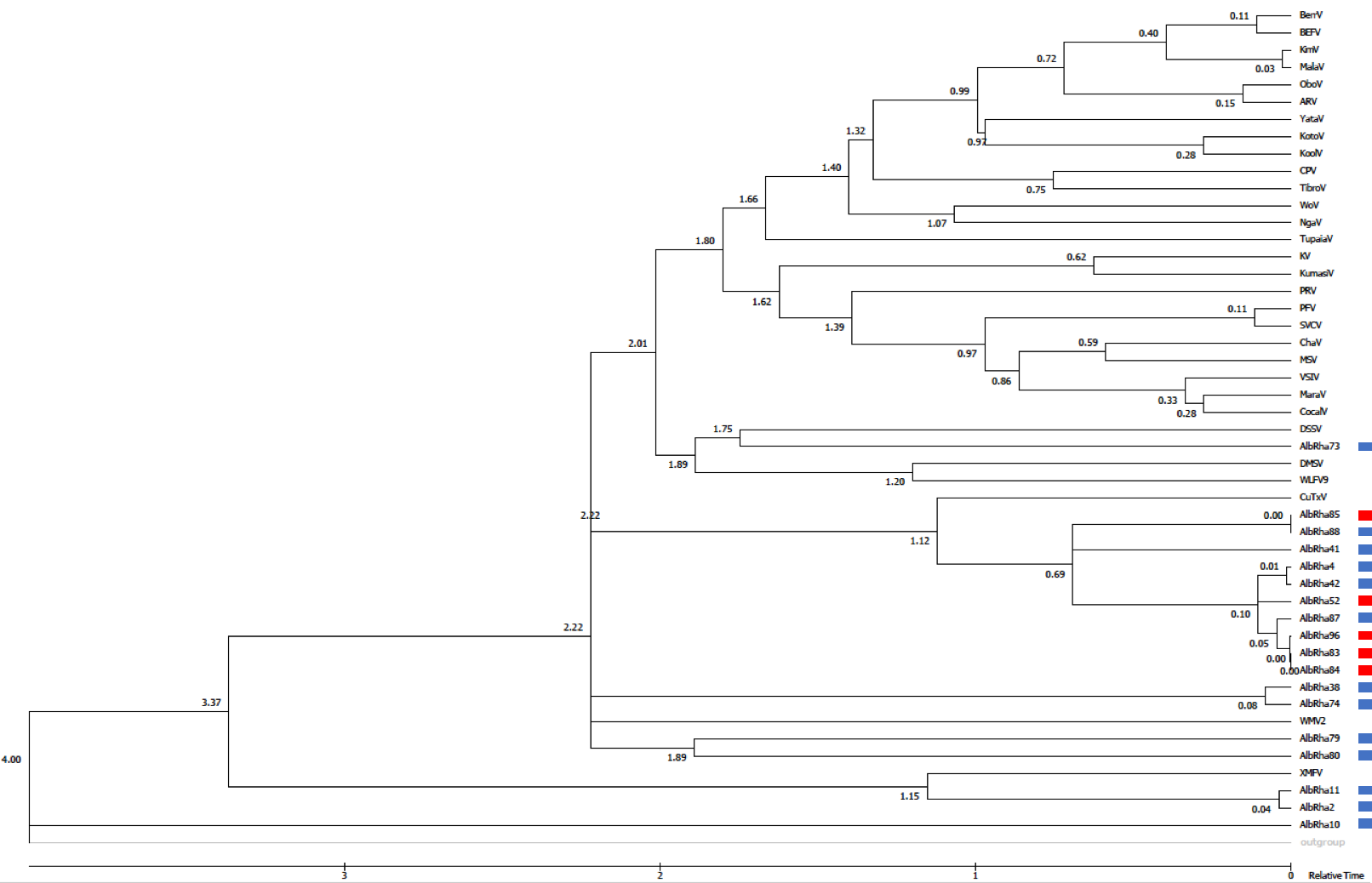


**(B) Rhabdovirus G protein**


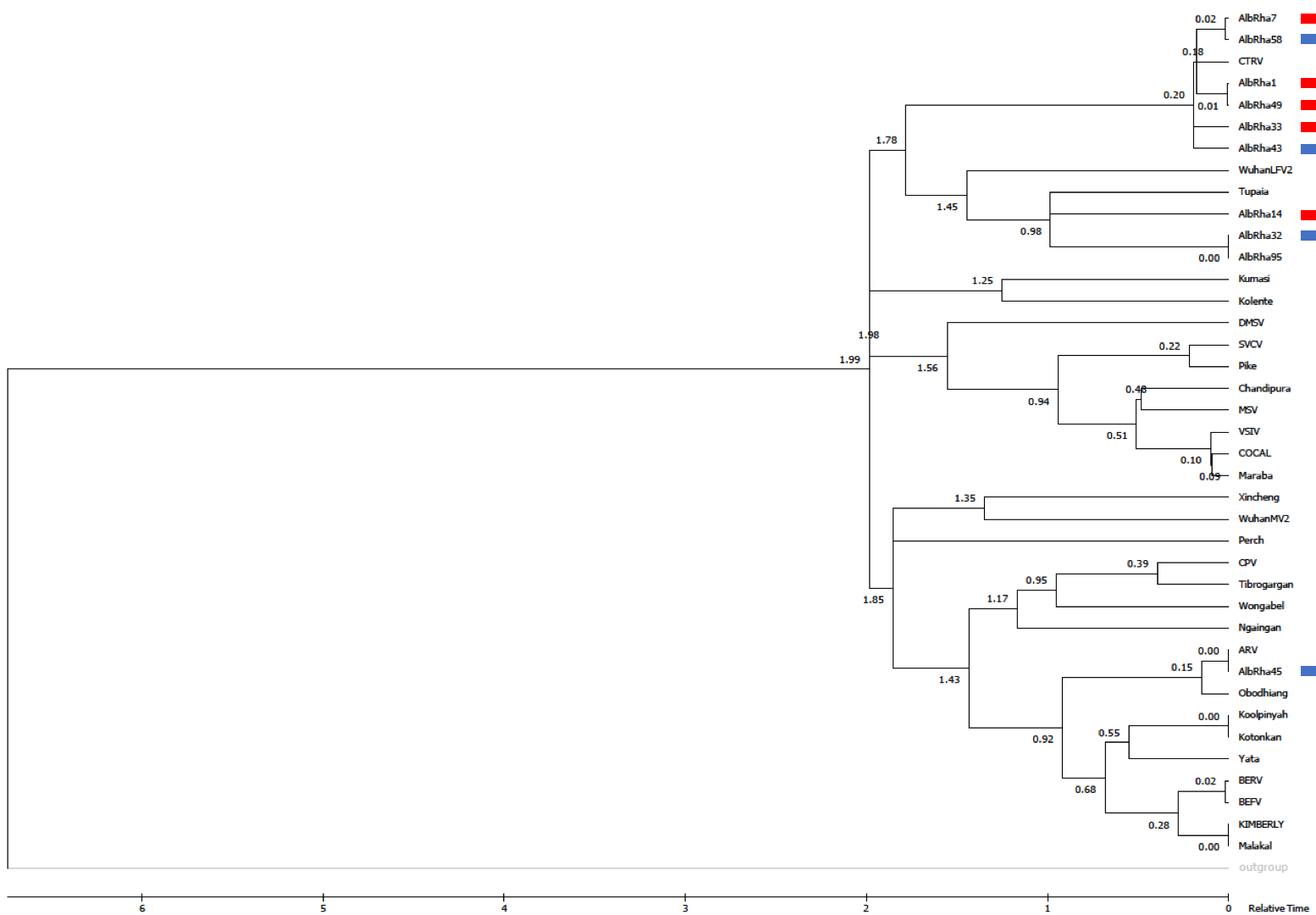


**(C) Flavivirus NS3 protein**


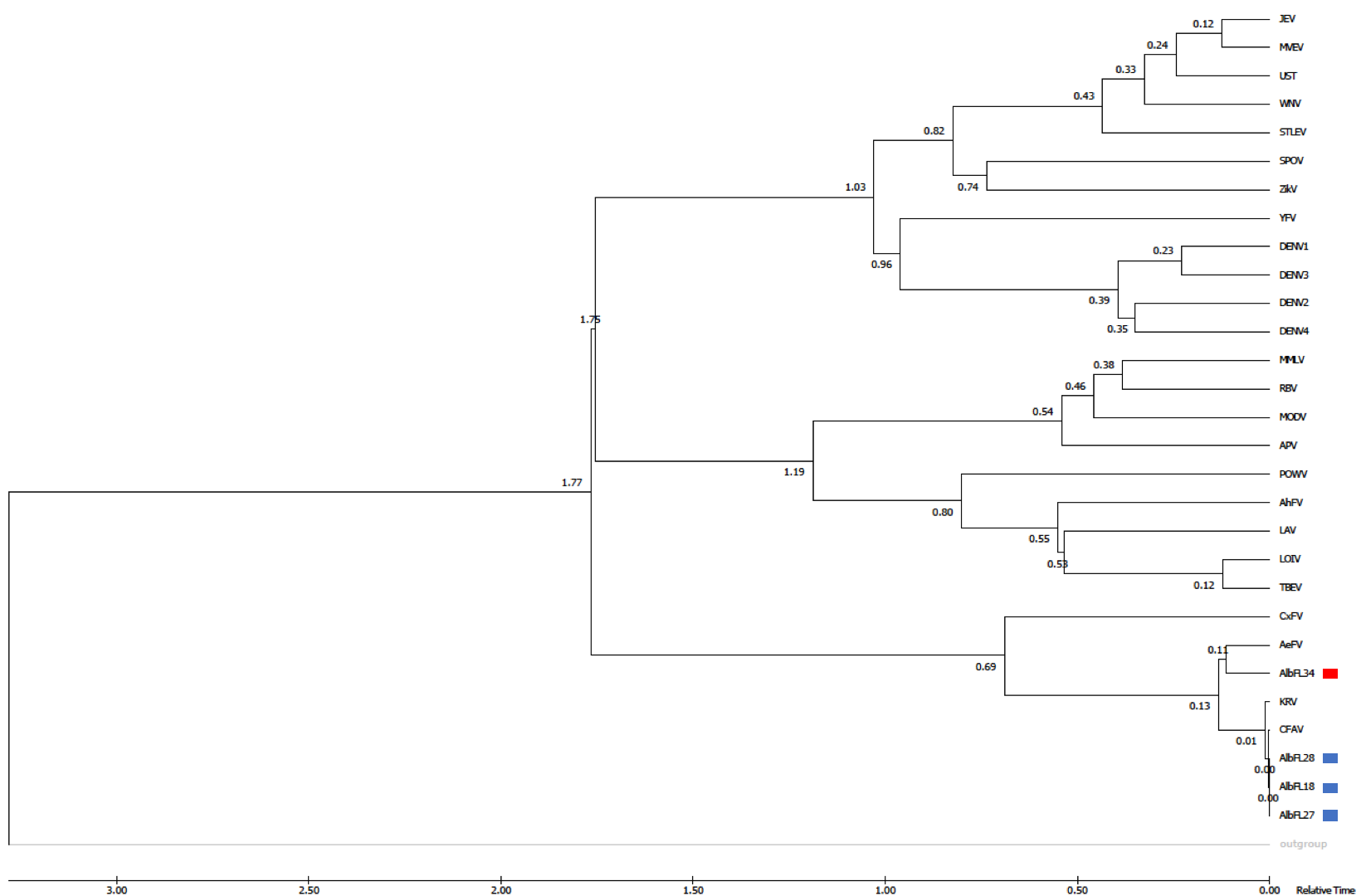


**(D) Flavivirus NS5 protein**


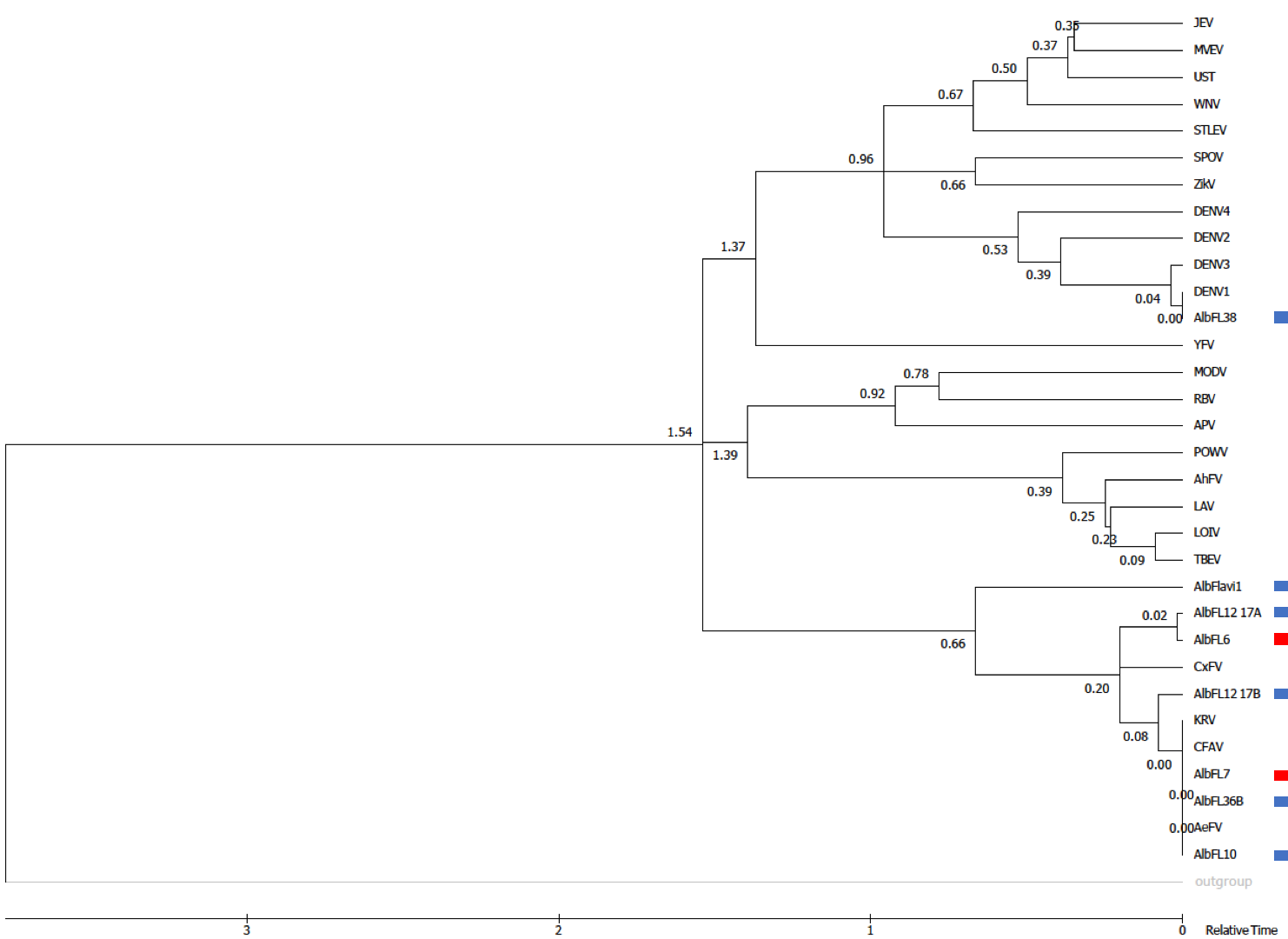


# Distribution of NIRVS, NIRVS genes and RNAi genes based on their LoP levels.

A-F letters indicate six different LoP classes. Grey lines are median LoP values of slow evolving genes (SGs) and fast evolving genes (FGs). F-NIRVS are blue, R-NIRVS are red, genes encompassing NIRVS are dark green (N-Gs), genes of the RNAi pathway are light green (R-Gs). Within F-NIRVS and R-NIRVS groups, shades of colors are used to highlight NIRVS mapping in exons of annotated genes, piRNA clusters or intergenic regions.


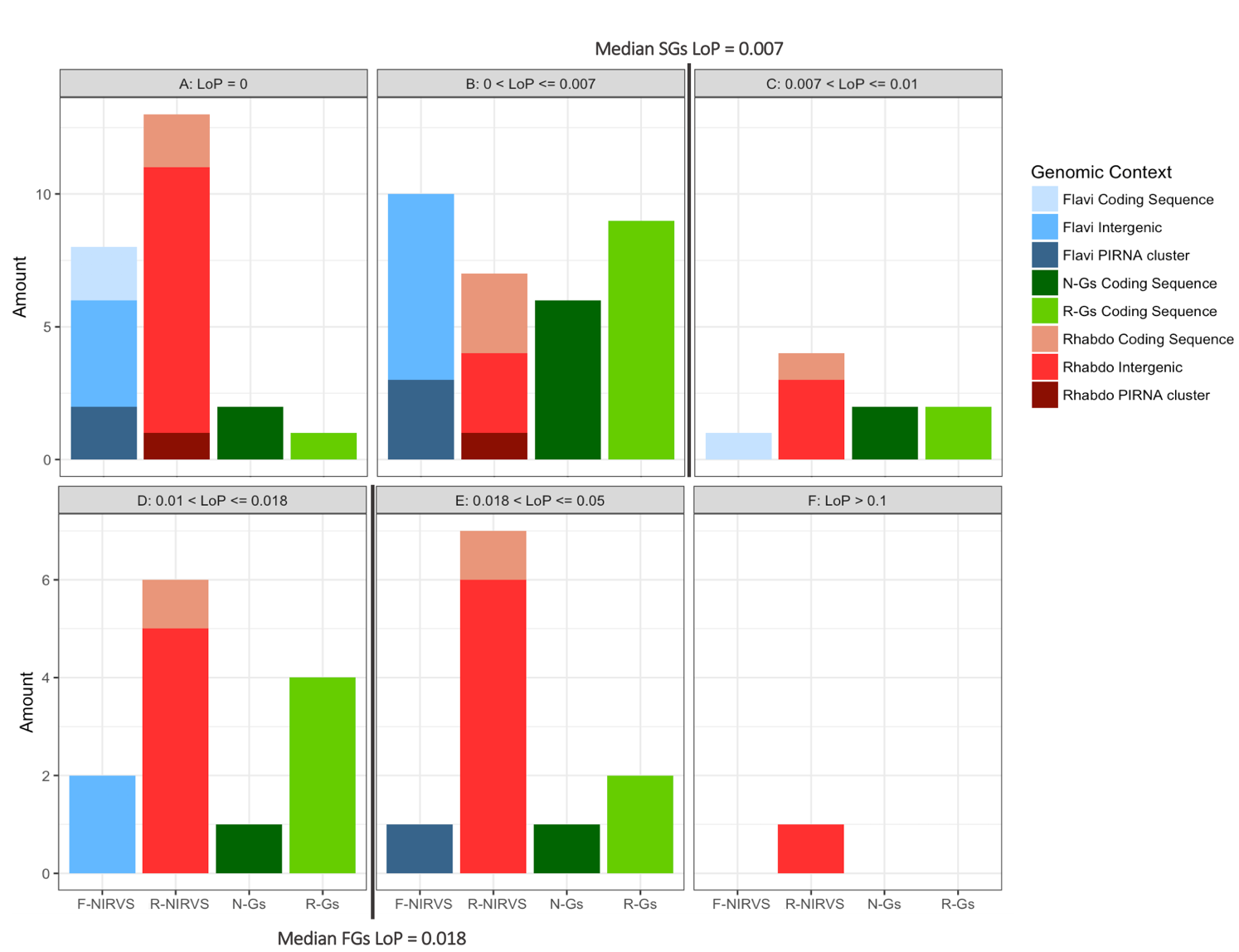

Supplement: Supplementary file 1 [file Data_Sheet_1.docx]
